# A Comprehensive Review of Radical-Mediated Intramolecular Cyano-Group Migration

**DOI:** 10.3390/molecules30142959

**Published:** 2025-07-14

**Authors:** Jia-Liang Zhu, Mei-Lin Chen

**Affiliations:** Department of Chemistry, National Dong Hwa University, Hualien 97401, Taiwan

**Keywords:** intramolecular migration, translocation, radicals, cyano group, nitrile, site-selective functionalization, photocatalysis

## Abstract

The radical-mediated intramolecular translocation of cyano groups has been recognized as a useful tool for the site-selective functionalization of organic molecules. The process is believed to proceed through the addition of an in situ-generated carbon-centered radical to the nitrile triple bond, followed by the β-scission of the resulting cyclic iminyl radical intermediate to relocate the cyano group and produce a more stable carbon radical for further elaboration. Beginning in the early 1960s and continuing for the next forty years, the research in this particular area has seen a surge of growth during the past two decades with advancements in radical chemistry and photocatalysis. The present article attempts to conduct a comprehensive review of existing studies on this topic by covering the literature from 1961 to 2025. The procedures developed for the purpose are grouped and discussed in four sections according to the strategies used to generate the initial carbon radicals, which include (i) hydrogen-atom transfer (HAT), (ii) radical addition to the π system, (iii) halogen-atom transfer (XAT), and (iv) the homolytic fission of a C-C single bond. In each section, a specific emphasis will be placed on reaction conditions, substrate scopes, and mechanisms.

## 1. Introduction

The cyano group is one of the most versatile functional groups in organic chemistry [1], which can be converted into carbonyl [2,3,4,5], amino [5,6,7], or heterocyclic [5,8,9,10,11] functionalities under suitable conditions. It can also serve as a directing, stabilizing, activating, or leaving group in many transformations, such as C-H functionalization [12], α-functionalization [13], Diels–Alder cycloaddition [14,15], cyclopropanation [16], and transition-metal-catalyzed hydrodecyanation [17]. Additionally, the cyano group has been recognized as an important pharmacophore in the drug discovery field, and it is often required to be incorporated into the structures of lead compounds for improving pharmacological properties or combating drug resistance [18,19]. The broad utility has promoted the development of numerous methods for the preparation of cyano compounds, which can be classified into several categories, according to the method of introducing the cyano group. One type of commonly used method is based on the conversion of a pre-installed functionality, e.g., an amide [20] or aldoxime [21], into the cyano group [22]. Another category of methods involves the cyanation of various substrates with external cyano-containing reagents, such as trimethylsilyl cyanide (Me_3_SiCN) [23], *N*-cyano-*N*-phenyl-*p*-toluenesulfonamide (NCTs) [24], butyronitrile [25], CuCN [26], or K_4_[Fe(CN)_6_] [27], that typically act as nucleophiles [23], electrophiles [24], or donors of CN ligand [25,26,27]. In some cases, NaN_3_ or TMSN_3_ was instead used as the nitrogen source for preparing aryl, alkenyl, or oxo-nitriles through the C-H or C-C bond cleavage of the corresponding hydrocarbons [28]. However, these methods are generally not applicable for the selective installation of the cyano group at an unactivated and/or a sterically congested carbon site within a molecule. To this end, one may resort to another approach known as intramolecular cyano-group migration (or translocation), which is usually implemented with readily available nitrile substrates and has proven to be particularly useful for preparing cyano compounds that are often non-trivial to obtain by conventional methods.

**Scheme 1:**
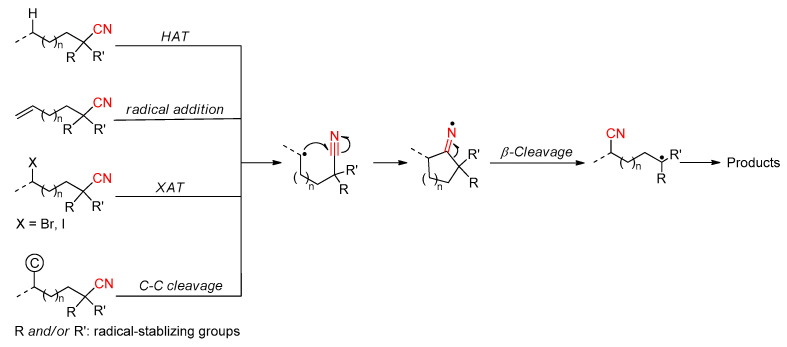
General schemes for radical-mediated cyano-group migration.

The radical–mediated translocation of cyano groups has emerged as a powerful tool for the site-selective functionalization of organic molecules. The process is believed to proceed through the addition of an in situ-generated carbon radical to the nitrile triple bond, followed by the β-cleavage of the resulting cyclic iminyl radical to relocate the site of the cyano group (Scheme 1). The β-cleavage step is usually driven by the formation of a more stable carbon-centered radical, which then undergoes various transformations to yield products. There are four general strategies that can be used to generate the initial carbon radical, including (i) hydrogen-atom transfer (HAT) from C(sp^3^)-H scaffolds; (ii) radical addition to an unsaturated π system, (iii) halogen-atom transfer (XAT), and (iv) homolytic fission of a C-C single bond. The research in this particular area begun in the early 1960s [29,30], and it benefited tremendously from advances in the fields of radical chemistry and photocatalysis over the past twenty years (before 2000: 18 publications; 2000 to now: 49 publications). This article intends to provide a comprehensive review of existing studies on this topic by covering the literature from 1961 to 2025. The procedures developed for the purpose will be discussed in four sections according to the strategies for producing the initiating carbon radical, with specific emphasis on the mechanism and substrate scope. Some research works discussed herein were also mentioned in several published accounts, but only as a small part of reviewing the migrations of a large array of functional groups [31,32,33,34,35,36]. Moreover, the non-radical methods reported for CN migration, mostly involving the release and recombination of cyanide anion or the formation of cyanide complex, are not included in this article.

## 2. Radical-Mediated Translocation of Cyano Groups

### 2.1. Site Selectivity in CN Migration

**Figure 1:**
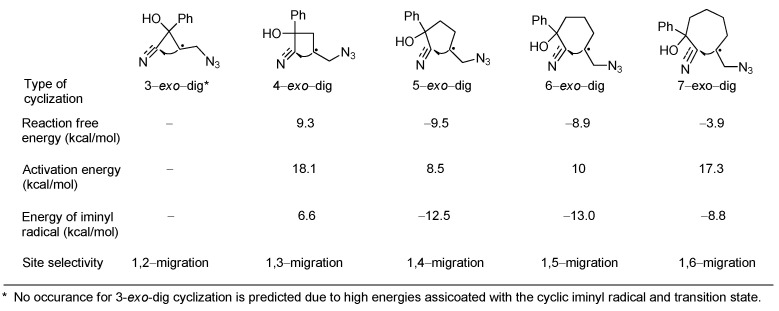
DFT energy calculation for radical cyclization onto nitrile group (Lafzi, 2023) [37].

Before we discuss the detailed synthetic strategies, it is instructive to clarify the origin of the site selectivity regarding the migratory distance of a cyano group from one carbon center to another. After the generation of the initial carbon radical, the site selectivity will be directed by the type of subsequent cyclization onto the nitrile group. It has been realized that the 1,4- or 1,5-cyano-migration caused by a 5- or 6-exo-dig cyclization is generally preferred over other migration processes, e.g., 1,3-, or 1,2-migration [34,35]. Lafzi previously reported a density-functional theory (DFT) study on the radical-addition triggered cyano-migration of alkenyl cyanohydrins, which can be used to illustrate this trend [37]. After the addition of the azido radical to C-C double bonds, the relative free energies calculated for the cyclization step are outlined in Figure 1. The calculations suggest that 3- or 4-exo-dig cyclization accounting for the 1,2- or 1,3-migration is either impossible or formidable due to the high energy associated with the three- or four-membered cyclic radical intermediate (or transition state), whereas 5- or 6-exo-dig cyclization is energetically favored to produce the corresponding cyclic iminyl radical. The energy profile of the 7-exo-dig cyclization appears to be more appealing when compared with that of the 3- or 4-exo-dig cyclization. However, the 1,6-cyano-migration resulting from the very process is rare in the literature, possibly due to an unfavorable distance between the reaction centers for intramolecular cyclization. It is also noteworthy that, although 4-exo-dig cyclization is generally disfavored, 1,3-cyano-migration has been achieved in several studies by using specific substrates and/or reaction conditions, as is explained in the following sections.

### 2.2. CN Group Translocation via HAT

**Scheme 2:**
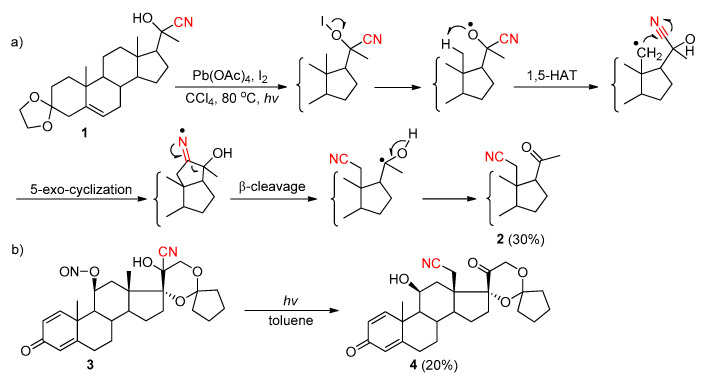
1,4-cyano migrations of steroidal cyanohydrins via HAT (**a**) with 20-hydroxy-20-cyano-steroid (**b**) with 11β-nitrite steroidal cyanohydrin (Kalvoda, 1961 and 1970) [29,38].

In 1961, Kalvoda reported the cyanohydrin-ketonitrile rearrangement of steroid **1** as the first known example to demonstrate the migratory aptitude of the cyano group (Scheme 2a) [29]. Mechanistically, the generation of an alkyoxy radical under the Barton hypoiodite reaction conditions triggered a 1,5-HAT, allowing the creation of a carbon radical from unactivated C(sp^3^)-H bond in the C-18 angular methyl group. Subsequent 5-exo cyclization onto the nitrile, followed by the β-scission of the resulting iminyl radical, provided a carbon-centered radical stabilized by the adjacent OH group. A further loss of a hydrogen-atom from this intermediate gave the rearrangement product **2**. About ten years later, the author described that the 1,4-cyano-migration could also be initiated via the N-O cleavage of the 11β-nitrite group in **3**, giving the formation of the product **4** upon irradiation (Scheme 2b) [38].

**Scheme 3:**
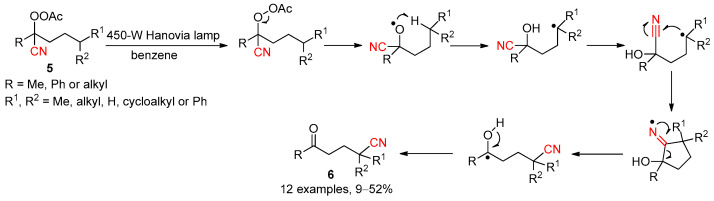
Conversion of α-peracetoxynitriles into δ-ketonitriles via HAT-mediated 1,4-CN migration (Watt, 1976) [39].

A similar cyano-migration was reported by Watt in 1976, after performing photolysis experiments on α-peracetoxynitriles **5** (Scheme 3) [39]. The reaction was initiated via the generation of an alkoxy radical through the homolytic cleavage of the O-O bond and afforded the δ-ketonitrile products **6** in low to moderate yields. Under the employed conditions, the competitive decarboxylation of **5** and/or Norrish type II fragmentation of **6** mainly accounted for the low yields encountered in most examined cases. Watt later explained that this photolytic protocol was comparable to Kalvoda’s method when applied to a steroid system, like delivering **2** in 21% yield from the corresponding 20-peracetoxy-20-cyanosteroid [40]. The Kalvoda and Watt procedures have not found much application since they were reported [41], possibly due to the inconvenience of preparing substrates and the lower yields associated with harsh reaction conditions. Despite this, these pioneering studies had provided valuable mechanistic insight into the migration process, thus creating a foundation for the future studies.

Photocatalysis, such as metal-based photoredox catalysis and organo-photocatalysis, has aroused great attention over the past two decades as a platform for the development of new synthetic strategies [42]. The photocatalysts, such as the commonly employed polypyridyl ruthenium or iridium complexes, can pass to excited states after absorbing light in the visible region of the electromagnetic spectrum. The excited species then interacts with organic molecules through energy transfer or single-electron transfer (SET), generating reactive intermediates, such as radical, radical anion, or diradical, to perform organic transformations. The excited catalysts will fall back to the ground state via unimolecular or bimolecular chemical processes, to complete a photocatalytic cycle.

**Scheme 4:**
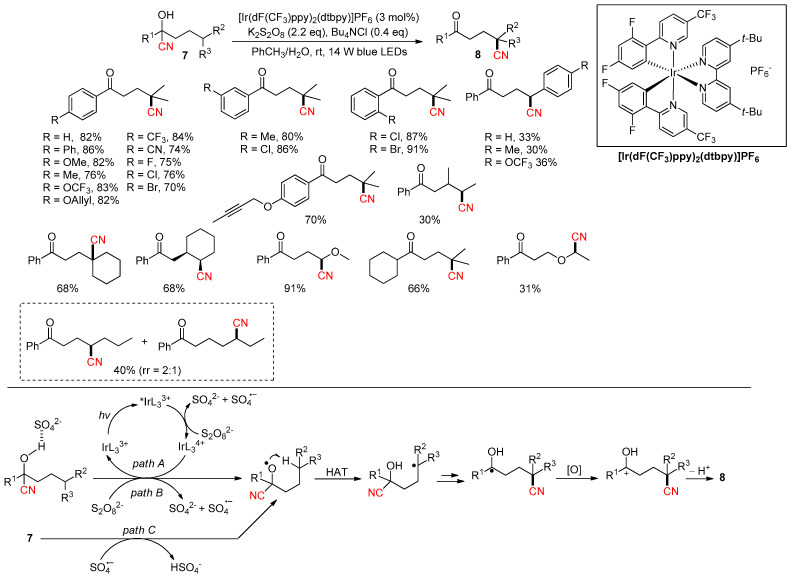
Conversion of cyanohydrins into δ-ketonitriles under photoredox catalytic conditions (Zhu, 2019) [43].

Photocatalysis has also been widely used for functional group translocations [32,36], with some studies being directed to HAT-mediated CN migration. In 2019, Zhu and co-workers reported a photoredox protocol for the direct conversion of cyanohydrins **7** into the corresponding δ-ketonitriles **8** without any pretreatment (Scheme 4) [43]. During the model study, the Ir(III) photocatalyst, [Ir(dF(CF_3_)ppy)_2_(dtbpy)]PF_6_, combined with K_2_S_2_O_8_ oxidant and Bu_4_NCl, were identified to be optimal among the tested variants, giving the best yield of the desired product upon visible-light irradiation (82%). The reaction was applicable to a range of cyanohydrins containing various functional groups, leading to the desired products in moderate to good yields. Additionally, the selective generation of the δ-ketonitrile over its ε-regioisomer was observed in the presence of multiple reactive methylene sites (rr: 2:1), owing to the fact that the six-membered cyclic transition state of 1,5-HAT is kinetically more favorable.

The process is initiated via the photoexcitation of IrL_3_^3+^ catalyst to *IrL_3_^3+^ that can be further oxidized through persulfate, giving IrL_3_^4+^, a sulfate ion (SO_4_^2−^), and a sulfate radical anion (SO_4_^•−^) [44]. The IrL_3_^4+^ species then converts the hydroxyl group into an oxy radical via a proton-coupled electron transfer (PCET) process, which is probably facilitated by the interaction with a conjugate base, e.g., a sulfate ion, to reduce the oxidation barrier of the O-H bond. This event also reduces the photocatalyst back to IrL_3_^3+^ to complete the catalytic cycle (pathway A). According to the report, the formation of the desired product, albeit at a lower yield, was still observed when the reaction was irradiated in the absence of a photocatalyst. The authors thus suggested that a persulfate-mediated chain reaction might concurrently engage in the PCET process to produce the alkoxy radical (path B). In addition, the possibility of abstracting the alcoholic hydrogen-atom via SO_4_^•−^ released from path A and/or B cannot be ruled out (path C). Subsequent 1,5-HAT generates a carbon radical, which then triggers the cyano-group migration, leading to a ketyl radical intermediate. Finally, the oxidation of this intermediate, followed by the deprotonation of the resulting carbocation, furnished the product.

**Figure 2:**
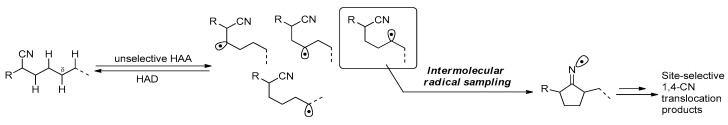
Concept of using radical sampling strategy to achieve 1,4-cyano translocation.

The alcohol in the aforementioned reactions did not take part directly in the CN transfer step, instead serving as an auxiliary to form the first carbon radical via HAT and to stabilize the second one. Overall, the migration of the cyano group was accompanied by the conversion of the hydroxyl group into a carbonyl and the loss of two hydrogen atoms. In a conceptually distinct contribution, Xu et al. recently reported an elegant photocatalytic, reversible C-H sampling strategy that enables the direct positional exchange between a CN group and an unactivated C-H bond without introducing any other variations to a molecule (100% atom economy) [45,46]. The transformation is conducted by merging hydrogen-atom donation (HAD) catalysis with hydrogen-atom abstraction (HAA) catalysis in one system, and it begins with the non-specific cleavage of C-H bonds via HAA to generate a series of carbon radicals (Figure 2). The resulting radicals are then sorted by the delicately established kinetic reversibility and the rate differences for the consequence step, permitting the C-4 radical to cyclize onto the nitrile to form the five-membered iminium radical intermediate whereas restoring other unfunctionalized radicals to C-H bonds through HAD.

**Scheme 5:**
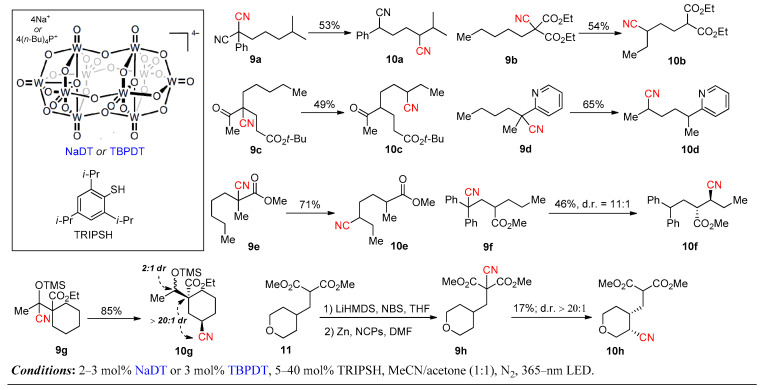
Selected examples for 1,4-CN migration of nitriles via radical sampling strategy (Xu, 2023) [46].

This strategy presents an unconventional paradigm to achieve site-selective C-H functionalization without requiring a site-selective C-H cleavage. As reported, a large array of malononitriles and mono-nitriles was smoothly converted to the desired 1,4-CN translocation products by using sodium or *tetra*-*n*-butylphosphonium decatungstate (NaDT or TBPDT) as a photo-HAA catalyst [47] and 2,4,6-triisopropylbenzenethiol (TRIPSH) as a HAD catalyst under 365-nm irradiation. Notably, the presence of a radical-stabilizing substituent at the α position (e.g., CN, Ar, CO_2_R, COR, or OTMS) is essential for CN migration. The selected examples in Scheme 5 demonstrate a remarkably highly functional group tolerance and site-selectivity between the 1,4- and 1,5-translocation (>20:1 r.r.) (**9a**–**g**→**10a**–**g**). Also outlined is a strategic sequence for the diastereoselective synthesis of compound **10h**, which involved the initial introduction of a cyano group to the readily accessible C-H site in **11**, followed by the migration of the CN group to the hard-to-target site in **9h**. Readers are encouraged to refer to the original paper for more interesting results and detailed discussions. More advances are expected in the future from the application of this “radical sampling” strategy to other systems.

### 2.3. Cyano-Group Migration via Radical Addition to Unsaturated CC Bonds

The addition of a radical species to a C=C double or triple bond can serve as another approach to triggering the cyano-group migration. The reactions in this category are abundant in the literature, and those reported from the late 1970s to the early 2010s are first discussed in this section.

#### 2.3.1. Early Reported Works

**Scheme 6:**
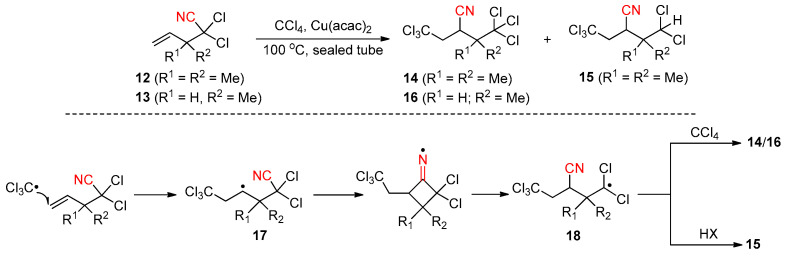
Trichloromethyl radical-triggered 1,3-cyano-migration (Johnson, 1982) [48].

The radical used for the addition step can be directly generated from a reagent. Johnson et al. first described that a trichloromethyl radical generated from CCl_4_ could add to the C=C double bonds of pent-4-enenitriles **12** and **13** to trigger a 1,3-cyano-migration and lead to the formation of the polychlorinated products **14**–**16** (Scheme 6) [48]. The driving force for the migration is the higher stability of the dichloroalkyl radical **18** than that of the secondary alkyl radical **17**. Moreover, the methyl substituents in the 3-position were found to be crucial for the reaction, possibly due to a Thorpe-Ingold effect in the cyclization step.

**Scheme 7:**
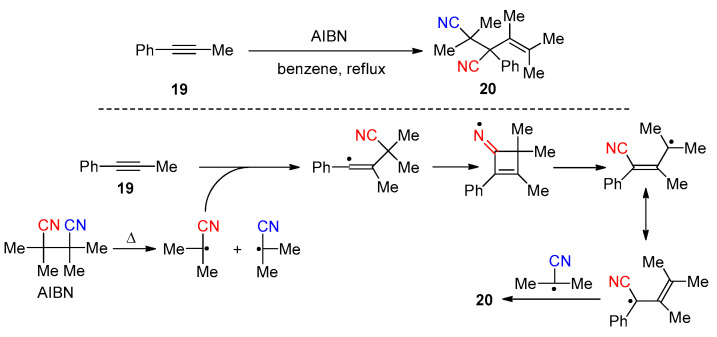
2-Cyano-isopropyl radical-triggered 1,3-CN migration (Montevecehi, 1997) [49].

Montevecehi et al. observed the formation of dinitrile compound **20** upon heating phenylpropyne **19** with 2,2′-azobis(2-methylpropionitrile) (AIBN) in benzene. The proposed mechanism involves the addition of 2-cyano-isopropyl radical (CIR) generated via the thermal decomposition of AIBN to the C≡C triple bond. The resulting vinyl radical then cyclizes onto the C≡N bond to form a cyclobutenyl iminium species. Subsequent ring opening with β-scission of the C-CMe_2_ bond affords a resonance-stabilized radical and thence **20** after trapping by another CIR (Scheme 7) [49]. Moreover, the cyclization of the vinyl radical appeared to be facilitated via the β-methyl group, as similar treatment on phenylacetylene (PhC≡CH) did not give any CN migration product.

**Scheme 8:**
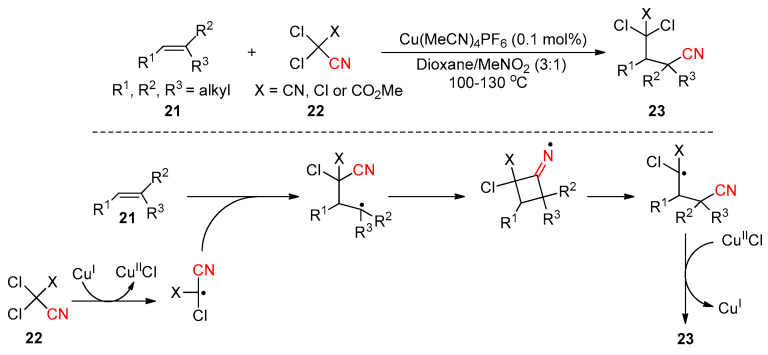
Radical-mediated carbocyanation of olefins (Inoue, 2012) [50].

In 2012, Inoue’s group reported a Cu(I)-catalyzed carbocyanation reaction of trisubstituted olefins **21** with chlorinated cyanides **22**, giving the formation of adducts **23** (11 examples, 24–78%) (Scheme 8) [50]. The process begins with the Cu(I)-promoted reductive C-Cl bond cleavage to form an electron-deficient carbon radical along with Cu(II)Cl. The addition of the radical to the C=C double bond from the less hindered side, followed by the cyclization of the resulting tertiary carbon radical onto the C≡N bond, produces a cyclic iminyl radical intermediate. Subsequent β-cleavage leads to a Cl-stablized radical through the 1,3-cyano-migration. Finally, abstraction of the chloride from Cu(II)Cl furnishes the products with regeneration of the Cu(I) catalyst. Notably, these reactions all involved 1,3-cyano-migration and consequently required elevated temperatures to overcome the high energy barrier associated with four-membered iminyl radicals and transition states.

**Scheme 9:**
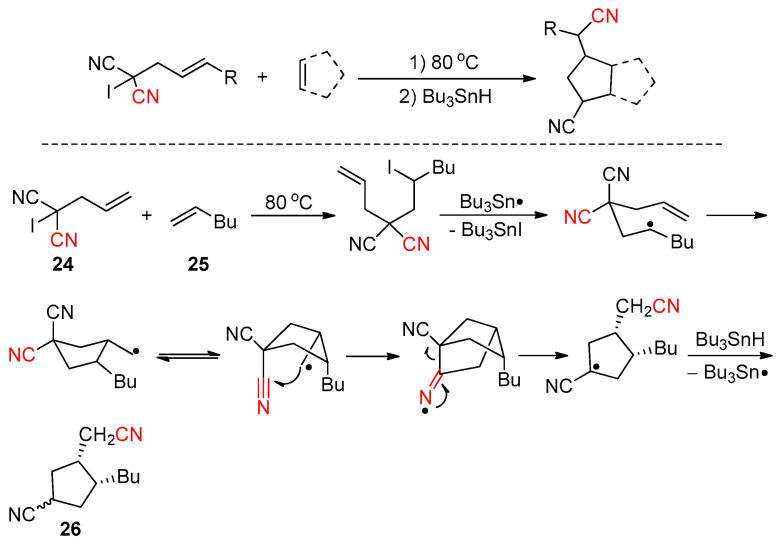
Annulation involving radical-addition-triggered 1,4-CN migration (Curran, 1992) [51].

**Scheme 10:**
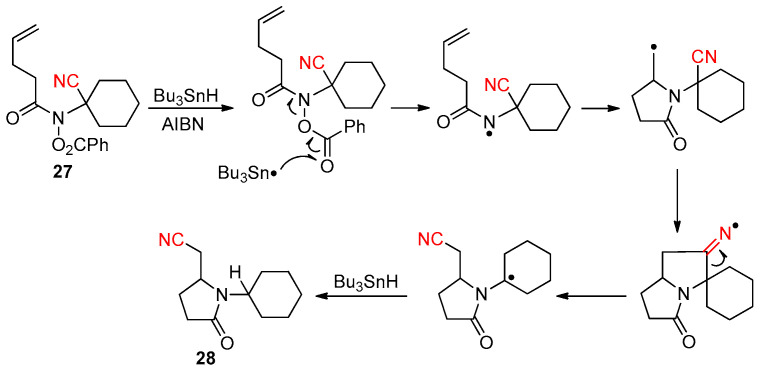
Amidyl radical-induced cyclization involving 1,4-CN migration (Zard, 1994) [52].

The development of cascade cyclization reactions through the CN migration strategy was also reported by using a reactive intermediate or a substrate itself as a radical precursor. Curran et al. reported an annulation reaction by heating iodomalononitriles such as **24** with alkenes, e.g., **25**, in the presence of tributyltin hydride (Scheme 9) [51]. The thermal coupling between **24** and **25** first occurs to form an iodane intermediate, which serves as a radical precursor and undergoes dehalogenation upon the action of Bu_3_Sn• to yield a secondary alkyl radical. The addition of the alkyl radical to the terminal alkene can trigger the 1,4-nitrile migration, leading to a tertiary carbon radical after 5-exo-dig-cyclization and β-cleavage. Finally, hydrogen-atom abstraction from Bu_3_SnH furnishes the product **26**. In this case, the β-cleavage is aided by relieving the ring strain of the bicyclic iminyl radical in addition to the radical-stabilizing nitrile group. Zard et al. developed another cyclization process terminating on a nitrile, in which the employed cyanide substrate **27** acts as a radical precursor via tributyltin radical-promoted N-O bond cleavage. The addition of the resulting amidyl radical to the olefin is followed by the cyclization and β-scission, yielding a tertiary carbon radical stabilized by the adjacent nitrogen atom and thence the product **28** after hydrogen-atom abstraction (Scheme 10) [52].

**Scheme 11:**
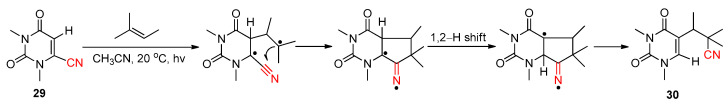
Formation of 5-substituted 1,3-dimethyluracil via the photoaddition of alkene to 6-cyanouracil (Saito, 1980) [53].

**Scheme 12:**
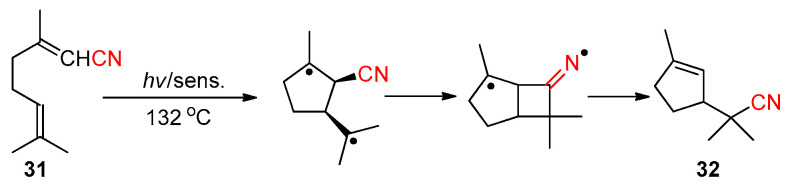
1,3-Migration of cyano group via photolysis of geranonitrile (Wolff, 1978) [54].

In two particular cases, the CN migration was proposed to proceed through a biradical intermediate. Saito and Matsuura reported the formation of 5-substituted uracil **30** upon irradiating 6-cyano-1,3-dimethyluracil **29** with 2-methyl-2-butene in acetonitrile (Scheme 11) [53]. The result was rationalized by assuming that a biradical intermediate resulting from the photoaddition of **29** with the alkene could engage in a 5-exo cyclization to afford a bicyclic iminium species. A subsequent 1,2-hydrogen shift, followed by β-cleavage, could furnish the rearranged adduct **30**. Furthermore, Wolff and Agosta described the photolytic conversion of geranonitrile **31** to the cyclopentene product **32** through 1,3-shift of the cyano group from a biradical intermediate (Scheme 12) [54]. This reaction was not observed at lower temperatures.

The above examples have demonstrated the diversity of substrates capable of undergoing radical-addition-induced cyano-migration. In recent years, such reactions have been mainly used to achieve difunctionalization of the unsaturated cyanohydrin or nitrile compounds. These studies, together with a few recently reported cyano-migration reactions involving diradical or radical cation intermediates, are discussed in the following sections, Section 2.3.2, Section 2.3.3, Section 2.3.4 and Section 2.3.5.

#### 2.3.2. Radical-Mediated Difunctionalization of Alkenes

**Scheme 13:**
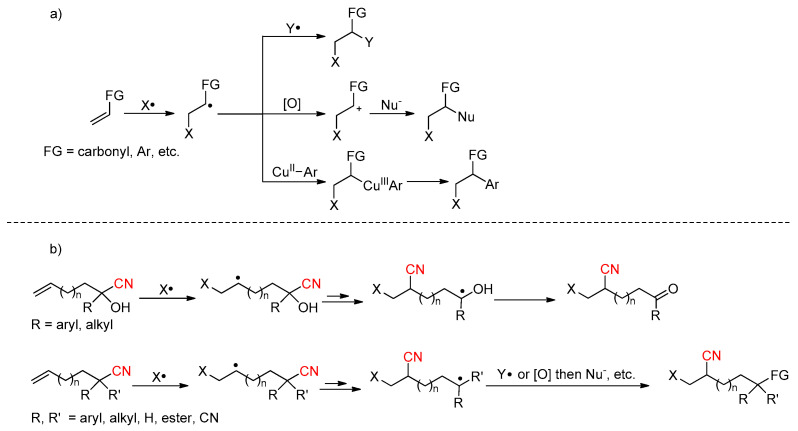
Strategies for radical-mediated alkene difunctionalization (**a**) via radical coupling, oxidation-nucleophilic addition or organometallation (**b**) via cyano-group migration.

Radical-mediated olefin difunctionalization has provided a powerful tool for enhancing the molecular complexity of simple olefins. In a common paradigm, the addition of a radical (X•) to the C-C double bond generates an alkyl radical, which can be trapped by another radical species (Y•) [55] or oxidized into a carbocation to facilitate the incorporation of second functional group via nucleophilic attack (Nu^−^) (Scheme 13a) [55,56]. In some cases, the carbon-centered radical can be sequestered by organometallic species [57,58] (e.g., Cu^II^-Ar) and functionalized through the reductive elimination of the resulting complex. However, the scope of alkenes in these transformations has been largely restricted to activated alkenes, including those containing aryl [55], carbonyl [59], or heteroatom [60] substituents that confer suitable electronic properties to the double bond for the incoming radical and stabilize the nascent carbon-centered radical through p-π conjugation. In comparison, the difunctionalization of unactivated alkenes (e.g., FG = alkyl) in a one-step fashion is more challenging and less explored [61,62]. Among several approaches to be considered, the radical-mediated distal functional group migration (FGM) has emerged as an attractive option, allowing the incorporation of diverse functionalities, such as the aryl, heteroaryl, carbonyl, alkynyl, alkenyl, imino, or cyano group, onto an alkene in addition to the initiating radical species [35,63,64]. Due to the pivotal roles of the cyano group in organic chemistry and related fields [1,2,3,4,5,6,7,8,9,10,11,12,13,14,15,16,17,18,19], direct cyanofunctionlization of olefin via the FGM strategy is unarguably an important subset of the alkene difunctionalization platform, which has been mostly performed with olefinic cyanohydrins or nitriles. During the processes, the ketyl radicals generated from cyanohydrins are readily oxidized to ketones, while the radical intermediates derived from olefinic nitriles undergo various transformations, such as radical coupling or oxidation to cations, followed by nucleophilic addition, to afford different kinds of products depending on reaction conditions (Scheme 13b). Despite this process being known for decades (Scheme 6), extensive studies on such transformations have only been carried out in the recent fifteen years with the flourishing development of radical chemistry and photocatalysis that enable efficient reagent systems to become increasingly available for the operation [63].

#### 2.3.3. CN Migration with Unsaturated Cyanohydrins

**Scheme 14:**
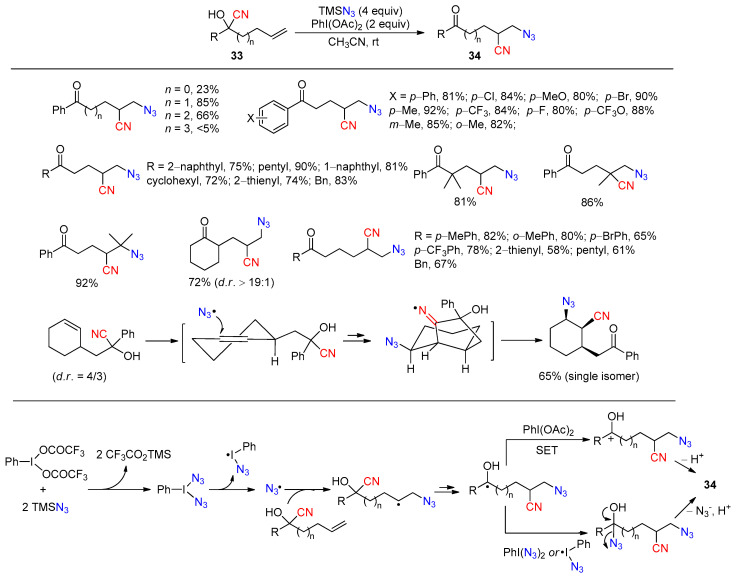
Radical-mediated azidocyanation of alkenes via 1,4(5)-CN migration (Zhu, 2016) [65].

The studies performed with unsaturated cyanohydrins are first discussed. In an early contribution, Zhu et al. reported a method for the azidocyanation of unactiavted alkenes by using TMSN_3_ and PhI(OAc)_2_ (PIDA) as the reagents (Scheme 14) [65]. During the initial assessment with the homologues cyanohydrins, the 1,4- and 1,5-nitrile transfers (*n* = 1, 2) were found to occur more efficiently than the 1,3- or 1,6-nitrile transfer reaction (*n* = 0, 3) as reflected in the yields of the products. In this context, a variety of bis- and trihomoallylic cyanohydrins **33** were further evaluated, giving the corresponding products **34** with complete regioselectivity. Particularly interesting is the generation of an all-cis-1,2,3-substituted single cyclohexane isomer from a 4:3 diastereomeric mixture. It would seem that this could be attributed to the facial selectivity in the radical addition step directed by the conformation of the cyclohexene ring and an equatorial orientation of the side chain. Mechanistically, the interaction of TMSN_3_ with PhI(OAc)_2_ produces PhI(N_3_)_2_, which then decomposes, giving an azido radical. The radical addition to the olefin, followed by 1,4(5)-cyano-migration, produces an α-hydroxy alkyl radical intermediate. As proposed, the conversion of this intermediate to the product may occur in two separate events: (i) PIDA-mediated single-electron oxidation to a carbonium ion, followed by deprotonation, or (ii) capture via the azido radical releasing from PhI(N_3_)_2_ or Ph(N_3_)I• [66], followed by the collapse of the resulting azidohydrin.

**Scheme 15:**
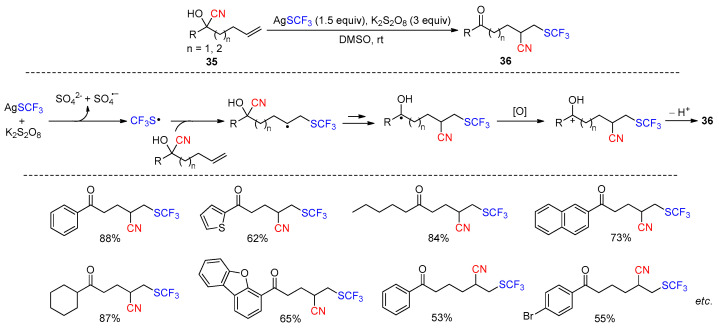
Radical-mediated cyanotrifluoromethylthiolation of alkenes via 1,4(5)-CN migration (Zhu, 2017) [67].

**Scheme 16:**
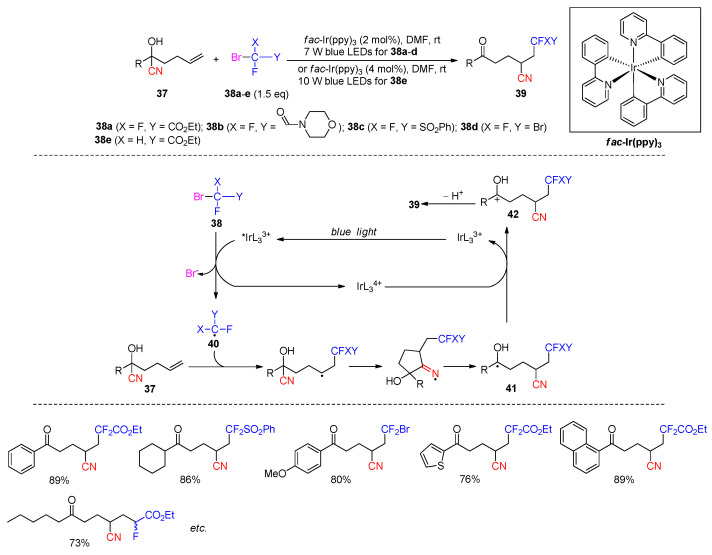
Synthesis fluorinated ketonitriles via photoredox-promoted 1,4-cyano-migration (Zhu, 2017) [68].

**Scheme 17:**
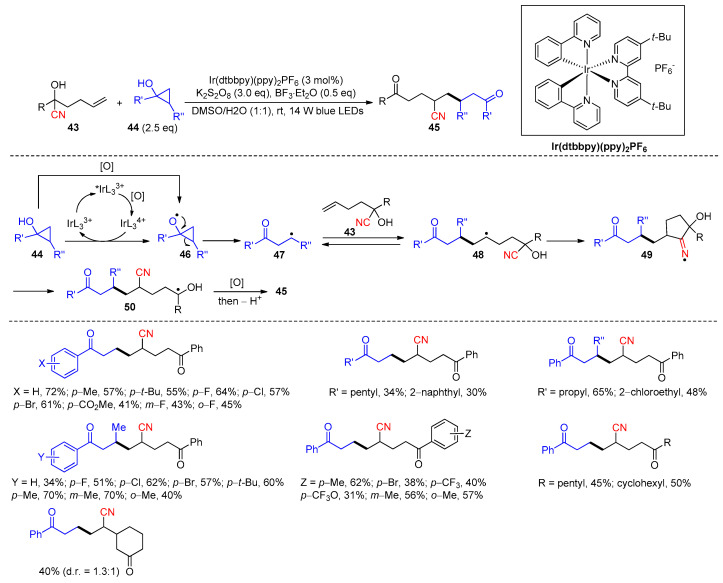
Synthesis of 1,8-diketones via photoredox-promoted coupling and 1,4-cyano migration (Zhu, 2019) [70].

**Scheme 18:**
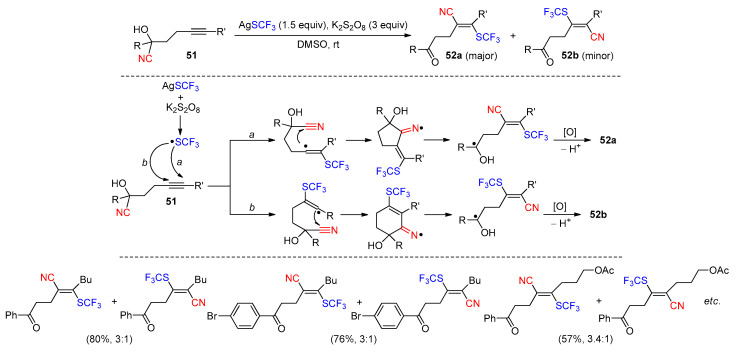
Cyanotrifluoromethylthiolation of alkynes via radical-mediated CN migration (Zhu, 2018) [71].

Subsequent to their initial report, Zhu and co-workers further disclosed several methods for the functionalization of unsaturated cyanohydrins using similar strategies (Scheme 15, Scheme 16, Scheme 17 and Scheme 18). In 2017, they reported the conversion of cyanohydrins **35** to CF_3_S-substituted ketonitriles **36** through the treatment with AgSCF_3_ and K_2_S_2_O_8_ (Scheme 15) [67]. In the process, trifluoromethyl sulfide is oxidized via persulfate to generate a trifluoromethylthio radical, which then adds to the alkene to trigger the cyano-migration and produce a ketyl-radical intermediate. The single-electron oxidation of this intermediate via persulfate, followed by the deprotonation of the resulting carbocation, affords the final product. Various substrates possessing the alkyl, naphthyl, thiophenyl, or dibenzofuryl group or phenyl rings with different substituents on the *ortho*, *meta*, and *para* positions afforded the corresponding products with moderate to good yields (26 examples, 35–88%).

Photoredox catalysis has also been utilized by Zhu’s group to achieve the polyfunctionalization of alkenyl cyanohydrins. In 2017, they reported the reaction of **37** with fluoro-substituted bromide reagents **38a**–**e**, including bromodifluoroacetate, 2-bromo-2,2-difluoro-1-morpholinoethanone, bromodifluoromethanesulfonylbenzene, dibromodifluoromethane, and bromofluoroacetate, for the synthesis di- or mono-fluorinated ketonitriles **39** (Scheme 16) [68]. Mechanistically, the iridium catalyst *fac*-Ir(ppy)_3_ (ppy = 2-phenylpyridine) (simplified as IrL_3_^3+^) engages in single-electron transfer with **38** from its photoexcited state, affording IrL_3_^4+^ and carbon radical **40** after the mesolysis [69]. The addition of **40** to **37**, followed by 5-exo-dig cyclization and β-cleavage, could cause the formation of ketyl radical **41**. Oxidation of **41** by the IrL_3_^4+^ species provides intermediate **42**, returning the catalyst to its Ir(III) oxidation state. Finally, the deprotonation of **42** furnishes the products. Various substrates bearing alkyl or aromatic substituents were allowed to react with **38a**–**e**, yielding over 40 products in good to high yields (50–91%). Moreover, increased catalyst loading and more intense radiation were required for the reactions of **38e** to improve the yields of mono-fluorinated products.

In another work, Zhu and co-workers described the preparation of 1,8-diketone derivatives **45** via the photoredox coupling between cyanohydrins **43** and cyclopropanols **44** (30 examples, Scheme 17) [70]. A reagent system comprising Ir(dtbbpy)(ppy)_2_PF_6_ catalyst, K_2_S_2_O_8_, and BF_3_Et_2_O was found to be optimal for the reaction, while the function of BF_3_·Et_2_O had remained unknown to the authors. In the process, the photoexcited catalyst *IrL_3_^3+^ is oxidized via K_2_S_2_O_8_ to yield IrL_3_^4+^ complex, which then converts **44** to the cyclopropyloxy radical **46** with the regeneration of the IrL_3_^3+^ catalyst. The formation of the desired product was still observed (albeit in a lower yield) when the reaction was conducted in the absence of a photocatalyst, suggesting that the oxidation of **44** via persulfate may concurrently contribute to the formation of **46**. The ring opening of **46** provides a β-carbonyl radical **47**, which then adds to the olefin to produce another alkyl radical **48**. The addition step may be reversible due to the similar thermodynamic stability of intermediates **47** and **48**. Nevertheless, the rapid interception of the carbon radical in **48** via the cyano group can drive the process forward to yield the iminium radical **49** and thence the more stable ketyl radical **50** after the β-cleavage. The single-electron oxidation of **50**, followed by deprotonation, gives the product.

Moreover, the 1,2-difunctionalization of internal alkynes was realized in a study by Zhu et al. It was found that cyanohydrins **51**, upon the treatment with AgSCF_3_ (1.5 equiv) and K_2_S_2_O_8_ (3 equiv), could be converted to the acrylonitrile products **52a** and **52b** as the mixtures of *E*-regioisomers (25 examples, **52a**/**52b** = 2:1 to 8:1, 40–83%) (Scheme 18) [71]. In the event, the addition of the in situ-generated CF_3_S radical occurs at both ends of the alkyne. The resulting alkenyl radicals then engage in the 1,4- or 1,5-cyano-migration to yield the ketyl radicals via cyclic iminium species. The oxidation of these intermediates, followed by deprotonation, leads to isomeric products. The ratios between **52a** and **52b** indicated that the 1,4-cyano-migration resulting from the outer-carbon addition (a) should occur more readily than the 1,5-migration triggered by the addition on the inner carbon atom (b).

**Scheme 19:**
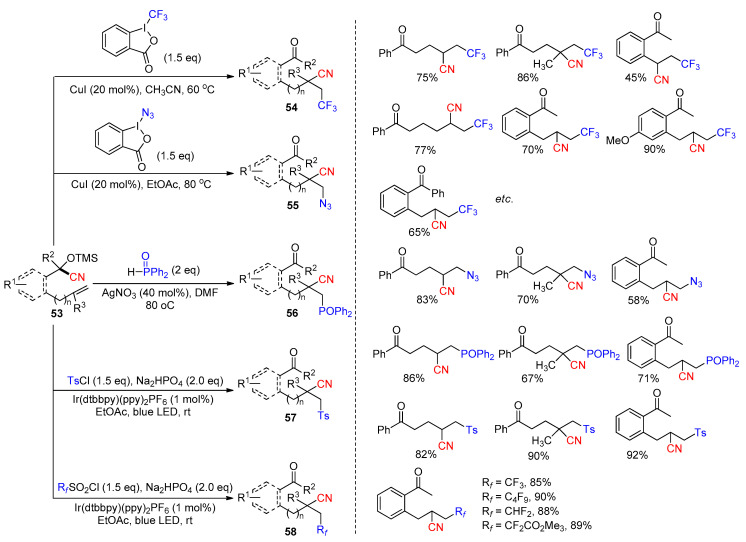
Diverse difunctionalization of alkenes via radical-mediated 1,4(5)-CN migration (Liu, 2016) [72].

The aforementioned studies by Zhu and co-workers have demonstrated the feasibility of achieving the 1,2-cyanofunctionalization of unsaturated CC bonds in cyanohydrins, given the efficient generation of carbon- or heteroatom-centered radicals to trigger the migration process. In addition to Zhu’s reports, the contributions from several other groups have also been noticed in the literature. In 2016, Liu et al. collectively reported a series of reactions of acyclic or aryl-tethered trimethylsilyl-protected cyanohydrines **53**, enabling the generation of the corresponding β-functionalized nitriles **54**–**58** via the trifluoromethyl, azido, phosphonyl, sulfonyl, or perfluoroalkyl radical-triggered 1,4(5)-cyano-migration, followed by oxidation and TMS-deprotection (Scheme 19) [72]. The trifluoromethylation or azidation reaction was conducted with 1-trifluoromethyl-1,2-benziodoxol-3(1*H*)-one (Togni reagent II) or azidobenziodoxolone (ABX) in the presence of CuI catalyst, while diphenyl phosphine oxide and AgNO_3_ were employed to effect phosphonylation. The sulfonylation and perfluoroalkylation were both performed under photoredox catalysis by using *p*-toluenesulfonyl chloride or fluorinated sulfonyl chlorides as radical sources. The mechanisms for the generation of initiating radical species, as well as the rationale for catalysis, albeit not being provided in the report, can be found in or deduced from other literature [73,74,75,76,77,78]. In a more recent report (2024), Li et al. similarly employed CF_3_SO_2_Cl as a radical precursor to achieve the trifluoromethylation of cyanohydrins, as in the production of **58** (R*_f_* = CF_3_) but without using a metal catalyst (455 nm Blue LED/diethyl ether/rt). The photo-induced C-Cl cleavage, followed by desulfonylation, was proposed to account for the generation of the requisite CF_3_ radical [79].

**Scheme 20:**
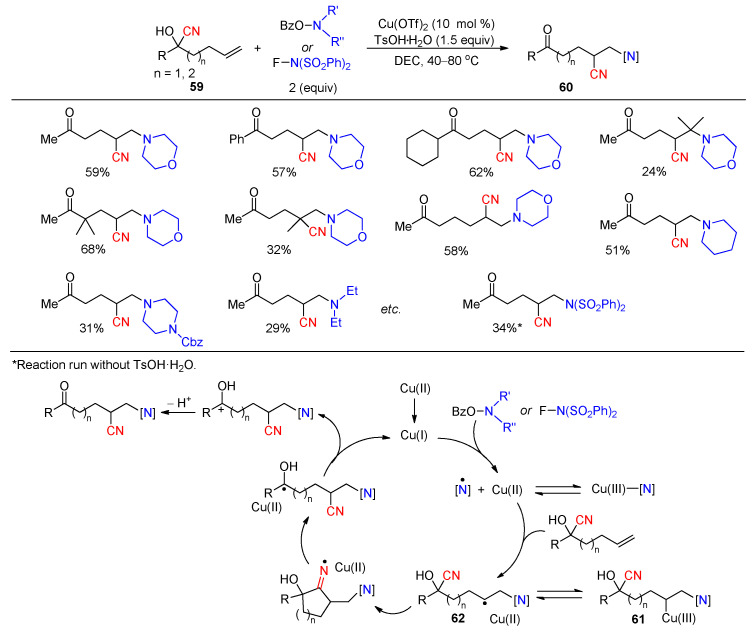
Copper-catalyzed 1,2-aminocyanation of alkenes via 1,4-CN migration (Wang, 2020) [80].

Wang and a co-worker developed another copper-catalyzed method for the 1,2-aminocyanation by using *O*-benzoylhydroxylamines and *N*-fluorobenzenesulfonimide (NFSI) as nitrogen precursors. The treatment of cyanohydrins **59** with these reagents in the presence of 10 mol% of Cu(OTf)_2_ and 1.5 equiv of TsOH afforded β-amino and β-sulfonimido nitriles **60** in varying yields (29 examples, 24–68%) (Scheme 20) [80]. The positive effect of *p*-TsOH on the formation of the desired product was observed but had not been fully understood by the authors. In the proposed mechanisms, the employed Cu(OTf)_2_ is first activated into the Cu(I) catalyst, presumably through the action of a nitrogen-containing ligand (*O*-benzyolhydroxylamine or NFSI) [81]. The resulting Cu(I) catalyst further engages in the oxidative addition with *O*-benzyolhydroxylamine or NFSI to generate an amino-Cu(III) complex. The addition of this complex to the alkene affords the complex **61**, which then undergoes reversible single-electron transfer to yield copper(II) and the secondary carbon radical **62**. Alternatively, intermediate **62** can be produced through the addition of an N-centered radical released from the amino-Cu(III) complex to the olefin. Subsequent cyano-migration through the cyclic iminium radical intermediate provides a more stable ketyl radical. Finally, SET oxidation of this radical by Cu(II) gives a carbocation and therein the product after the deprotonation, which also allows the regeneration of copper(I) catalyst for the next cycle.

**Scheme 21:**
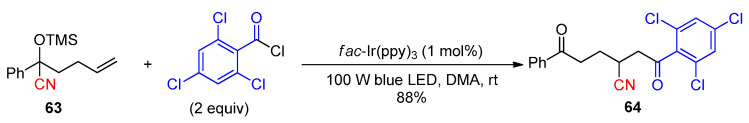
Benzoylcyanation of alkene via Photoredox-promoted 1,4-CN migration (Ngai, 2019) [82].

**Scheme 22:**
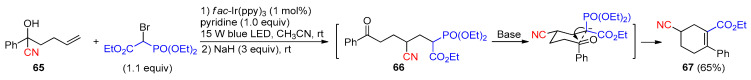
Construction of cyclohexene via radical-mediated cyanofunctionlization of alkene and HWE olefination (Feng, 2021) [83].

There are two other reported cases of photoredox reactions using *fac*-Ir(ppy)_3_ as a catalyst. As a part of investigating the distal migration of a variety of functional groups, Ngai et al. demonstrated that an aroyl radical, generated upon the reduction in 2,4,6-trichlorobenzoyl via the excited photoredox catalyst, could add to the alkene in the TMS-protected cyanohydrin **63** to trigger the 1,4-cyano-migration and cause the formation of the β-cyanated-1,6-diketone **64** after a further oxidation–deprotection sequence (Scheme 21) [82]. Feng et al. described the construction of cyclohexene **67** by merging the catalytic reaction between cyanohydrin **65** and 2-bromo-2-phosphorylacetate with the one-pot HWE olefination of the resulting product **66** (Scheme 22) [83]. The proposed pathway leading to **66** is similar to that shown in Scheme 16.

**Scheme 23:**
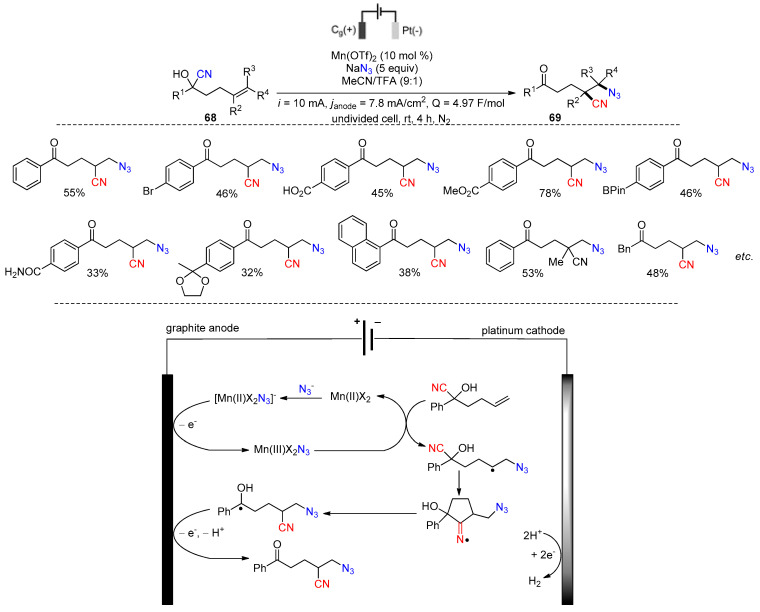
Electrochemical alkene azidocyanation via 1,4-nitrile migration (Morrill, 2022) [84].

The azidocyanation reaction shown in Scheme 14 utilizes PIDA as a high-molecular-weight oxidant, generating stoichiometric organic waste that requires separation from the desired products. In a conceptually distinct contribution, Morrill and co-workers developed an alternative oxidant-free protocol to achieve the azidocyanation of alkenes under electrochemical conditions. It was found that a system composed of Mn(OTf)_2_ and NaN_3_ as both an azide source and an electrolyte in MeCN/TFA was capable of converting aromatic (R^1^ = aryl) and aliphatic (R^1^ = alkyl) cyanohydrins **68** into the 1,2-azidonitriles **69** upon galvanostatic cycling (28 examples, 33–55%) (Scheme 23) [84]. An array of useful functional groups, such as halogen, carboxylic acid, ester, borane, amide, and ketal in the 4-position of a phenyl ring, were tolerated under the developed conditions, giving the potential for further derivatization. Based on the results obtained from the radical clock and cyclic voltammetry studies, the authors proposed that the process is initiated via the formation of [Mn(II)X_2_N_3_] from Mn(II)X_2_ and NaN_3_, which is oxidized at the anode to form Mn(III)X_2_N_3_. This intermediate may deliver an azide radical to the cyanohydrin, giving a secondary alkyl radical. After the 1,4-nitrile migration, a second oxidation of the resulting ketyl radical at the anode, followed by deprotonation, provides the final product. Meanwhile, hydrogen gas is evolved via a proton reduction at the cathode.

#### 2.3.4. CN Migration with Alkenyl Nitriles

Radical addition-triggered CN migration has also been widely used for the multi-site functionalization of alkenyl nitriles. The carbon-centered radicals resulting from the CN migration were shown undergo a variety of transformations in this category, which are classified and discussed in four sub-sections: (i) capture via radicals, organometallic species or phenyl ring, (ii) oxidation to carbocations, followed by nucleophilic addition, (iii) reduction to carbanions, followed by protonation or nucleophilic substitution, and (iv) the formation of CC double bonds via oxidation/deprotonation, elimination, or HAT.

##### Capture via Radicals, Organometallic Species, or Phenyl Ring

**Scheme 24:**
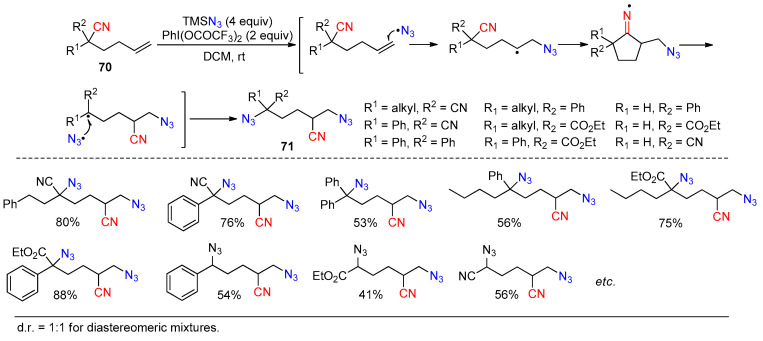
Trifunctionalization of hexenenitriles via azido radical-mediated cyano-migration (Zhu, 2022) [85].

First, the carbon radicals can be sequestered by external radical species, either identical to or different from those used for the addition step. Zhu et al. in 2022 reported the conversion of a variety of hexenenitriles **70** into the diazidation products **71** by using TMSN_3_ as the radical source and PIFA as a stoichiometric agent (30 examples, 41–90%) (Scheme 24) [85]. In the process, azido radicals should be generated in a similar way as shown in Scheme 14. After this, one azido radical triggers the 1,4-cyano-migration through the addition to **70**, while the other one intercepts the resulting carbon radical to give the product. Once again, the presence of at least one radical-stabilizing substituent (R^1^ and/or R^2^ = CN, Ph, CO_2_Et) is required to facilitate the β-C-C bond cleavage.

**Scheme 25:**
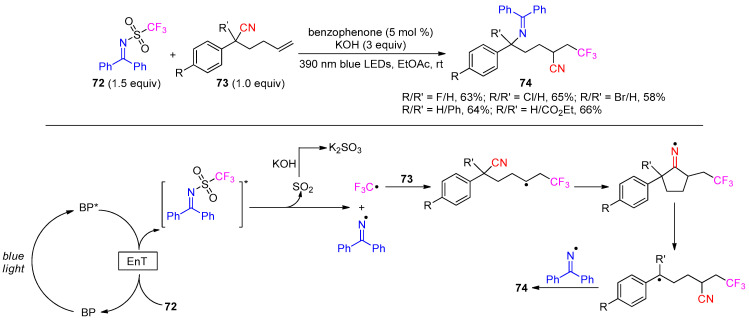
Photochemical alkene trifluoromethylimination involving 1,4-CN migration (Yang, 2023) [86].

Yang et al. have developed a photolytic protocol for the trifluoromethylimination of alkenes by using (diphenylmethylene)-1,1,1-trifluoromethanesulfonamide **72** as a bifunctional reagent. In the event, a benzophenone (BP)-mediated photosensitized energy transfer (EnT) promotes the successive N-S-C bond cleavage in **72** to generate a diphenyl iminyl and trifluoromethyl radical pair after the extrusion of SO_2_. The consecutive delivery of these radicals to an alkene provides the β-CF_3_ imine product. Moreover, KOH was used as an external base to remove the released SO_2_. When the reactions were performed with the alkenes **73** tethered to a cyano group, 1,4-CN migration occurred prior to the radical coupling to give the trifunctionalization products **74**, as shown in Scheme 25 [86].

**Scheme 26:**
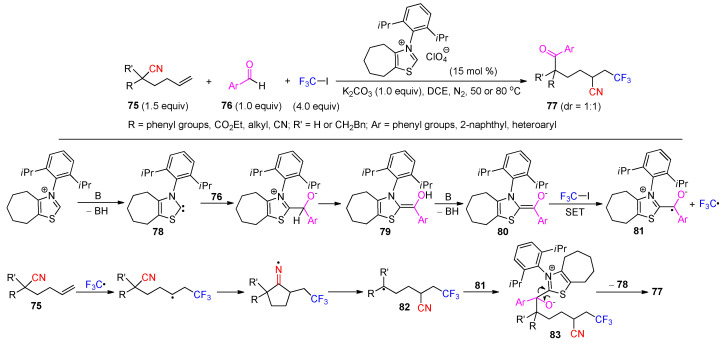
NHC-catalyzed radical-relay trifluoromethylation-acylation of hexenenitriles via cyano-migration (Du, 2023) [87].

The radical-mediated trifunctionalization of alkenyl nitriles has also been realized via *N*-heterocyclic carbene (NHC) organocatalysis. In 2023, Du et al. reported a NHC-catalyzed three-component reaction of hexenenitriles **75** with aromatic aldehydes **76** and trifluoromethyl iodide, which provided the products **77** (33 examples, 55–87%) by using a thiazolium salt as precatalyst and K_2_CO_3_ as base (Scheme 26) [87]. In the proposed pathway, the carbene **78** derived from the precatalyst reacts with **76** to form enaminol **79** (the so-called Breslow intermediate) and further enolate **80** after proton abstraction. A single electron transfer (SET) from **80 [88]** to CF_3_-I would produce NHC-bound ketyl anion radicals **81**, along with a trifluoromethyl radical. The subsequent addition of the CF_3_ radical to **75**, followed by the 1,4-CN migration, provides carbon radical **82**, which then undergoes the coupling with **81** to give intermediates **83**. Finally, the collapse of **83** leads to the product **77** and the regeneration of carbene **78** for the next cycle. In addition to CF_3_I, this radical-relay protocol was also applicable to several other fluorinated reagents, including BrCF_2_CO_2_Et, CF_2_Br_2_, and C_6_F_13_I, as well as ICH_2_Ts. However, it was not compatible with the tested aliphatic aldehyde as areplacement for **76**.

**Scheme 27:**
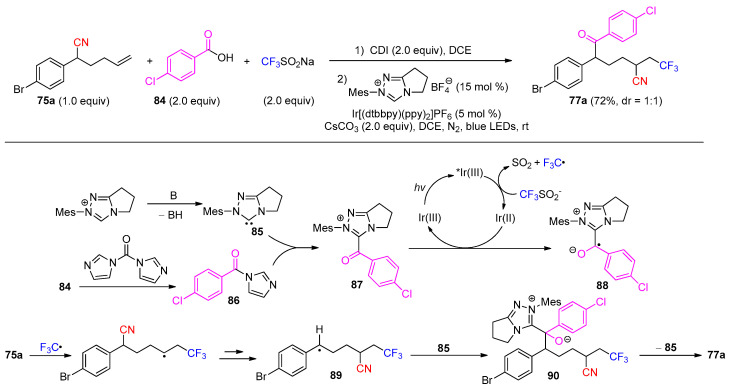
Dual NHC/photoredox catalytic trifluoromethylation-acylation of hexenenitriles via cyano-migration (Du, 2024) [89].

More recently, the same research group described an alternative method for the trifluoromethylation-acylation of hexenenitriles by instead using aromatic acids and CF_3_SO_2_Na as the reactants. Besides, a dual NHC/photoredox catalysis system comprising a triazolium salt, Ir(dtbbpy)(ppy)_2_PF_6_ catalyst, and Cs_2_CO_3_ was used to effect the transformation upon visible-light irradiation [89]. Additionally, the method also requires the pre-activation of the acids into the corresponding benzoylimidazoles through the in situ treatment with 1,1′-carbonyldiimidazole (CDI). As a typical example, the reaction of hexenenitrile **75a** with CF_3_SO_2_Na and acid **84** under the developed conditions afforded the product **77a** in good yield (Scheme 27). A likely mechanism involves the addition of NHC **85** to the carbonyl group of benzoylimidazole **86**, giving acyl azolium **87**. Meanwhile, the single-electron oxidation of CF_3_SO_2_Na via the excited catalyst produces a CF_3_ radical, along with the Ir(II) species that is sufficiently powerful to reduce **87**, thereby generating NHC-bounded radical anion **88**. The addition of the CF_3_ radical to **75a**, followed by cyano-migration, provides benzylic radical **89**, which can couple with **88** to yield a highly congested intermediate **90**. Finally, the decomposition of **90** furnishes the product **77a** with the regeneration of **85** to complete the cycle. 

**Scheme 28:**
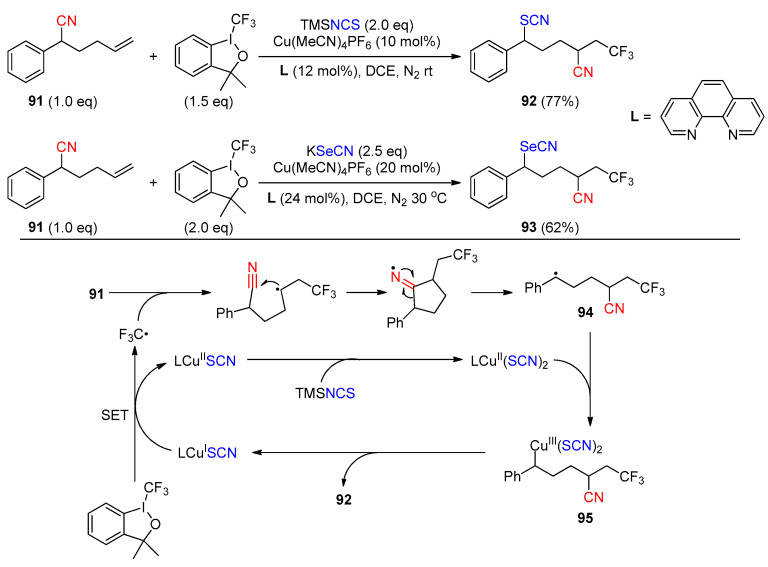
Copper-catalyzed 1,5-trifluoromethylthio- or selenocyanation of 5-hexenenitriles via cyano-migration (Guo, 2025) [90].

The carbon radical resulting from CN migration can also be intercepted by an organometallic species. In a recent report, Guo et al. described the remote 1,5-trifluoromethylthio- or selenocyanation of 5-hexenenitriles (e.g., **91**) with a cyano-migration through the copper catalysis, affording thiocyanates or selenocyanates (e.g., **92** or **93**) under the conditions shown in Scheme 28 [90]. In a proposed mechanism, the in situ-formed LCu^I^SCN undergoes a single-electron transfer (SET) with the employed trifluoromethyl-1,3-dihydro-3,3-dimethyl-1,2-benziodoxole (Togni reagent I) to generate LCu^II^SCN and CF_3_ radical. The latter then adds to **91**, triggering the 1,4-CN migration to form benzylic radical **94**. In the presence of TMSNCS, the LCu^II^SCN species can be converted to LCu^II^(SCN)_2_, which combines with **94** to give the alkyl-Cu^III^(SCN)_2_ intermediate **95**. This intermediate further undergoes a reductive elimination to provide the product and regenerate the LCu^I^SCN species.

**Scheme 29:**
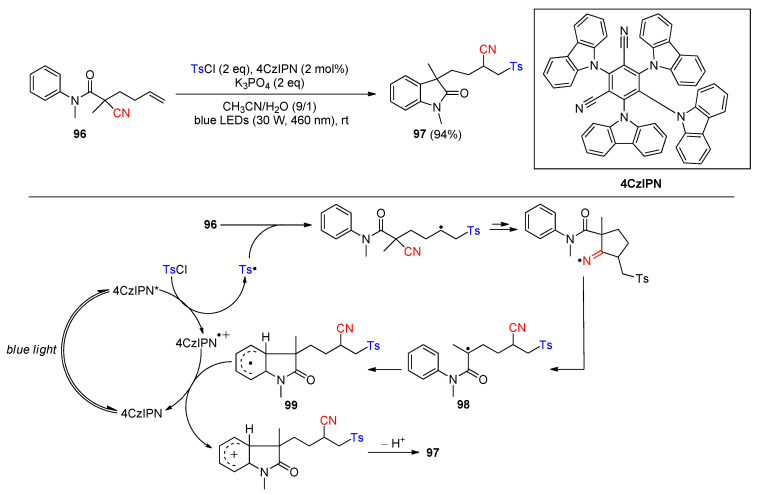
Construction of oxindoles via tandem sulfonylation-initiated cyano-migration/cyclization cascade (2025, Wang) [91].

Wang et al. have revealed an elegant synthesis of the functionalized oxindoles via a tandem sulfonylation-initiated cyano-migration/cyclization cascade under photoredox conditions [91], in which the carbon radical resulting from the CN migration was shown to be captured via a phenyl moiety. As an example, irradiation of enamide **96** and tosyl chloride (2 equiv) with 2,4,5,6-tetrakis(9*H*-carbazol-9-yl) isophthalonitrile photocatalyst (4CzIPN) (2 mol %) and K_3_PO_4_ (2.0 equiv) led to the desired product **97** in 94% yield (Scheme 29). Mechanistically, 4CzIPN can be transferred to its excited state through visible light, initiating an SET with TsCl to generate a sulfonyl radical and the 4CzIPN^•+^ species. The sulfonyl radical then adds to **96** to trigger CN migration and produce an alkyl radical **98**. The radical further undergoes intramolecular aromatic addition to form intermediate **99**, which is ultimately oxidized via 4CzIPN^•+^ to yield a carbocation and thence the product after the deprotonation.

##### Oxidation to Carbocations, Followed by Nucleophilic Addition

**Scheme 30:**
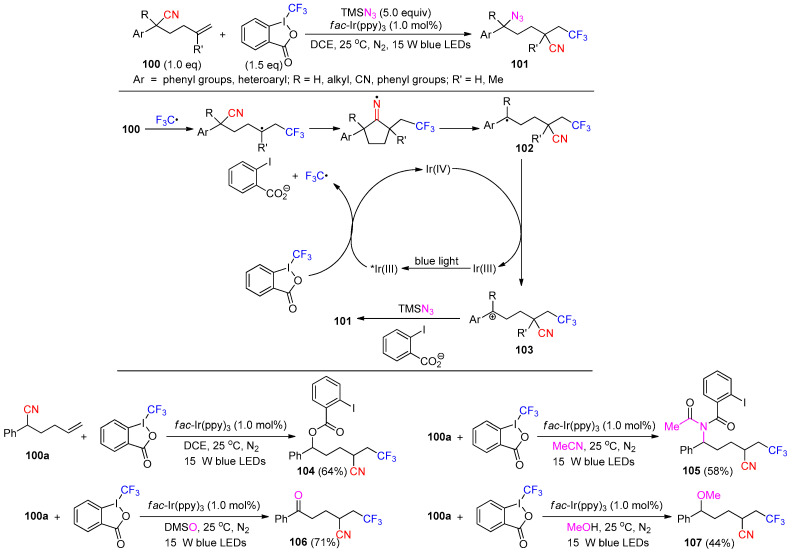
Photoredox functionalization of hexenenitriles with Togni II reagent and external nucleophiles (Chen, 2022) [92].

The carbon radical resulting from CN migration can be oxidized into a carbocation, thus offering an opportunity for the incorporation of the second functional group via a nucleophilic attack. Chen and Zhu in 2022 reported the photoredox trifunctionalization of 5-hexenenitriles **100** using Togni reagent II as radical source and TMSN_3_ as a nucleophile precursor. The reaction was conducted with a *fac*-Ir(ppy)_3_ catalyst to provide the polyfunctionalized products **101** upon visible light irradiation (31 examples, 32–86%) (Scheme 30) [92]. In the process, the reduction in the Togni reagent via the excited Ir(III) catalyst gives a trifluoromethyl radical and 2-iodobenzoate. The addition of the CF_3_ radical to **100** triggers the 1,4-CN migration to provide a benzyl radical **102** after the cyclization and β-cleavage. Then, the single-electron oxidation of **102** by Ir(IV) species produces carbocation **103** with the regeneration of the ground state Ir(III) catalyst. Finally, the nucleophilic attack of the azide anion on **103** furnishes the product. It was additionally found that the reaction could proceed with other external nucleophiles. In the absence of TMSN_3_, the Togni reagent served as both the radical source and the nucleophile precursor to give the ester product **104**. When MeCN was employed as the solvent, a Ritter-type addition dominated to yield the amidation product **105** via subsequent nucleophilic attack with 2-iodobenzoate and Mumm rearrangement. DMSO and MeOH were also attempted as nucleophiles, leading to the formation of the products **106** and **107**, respectively.

**Scheme 31:**
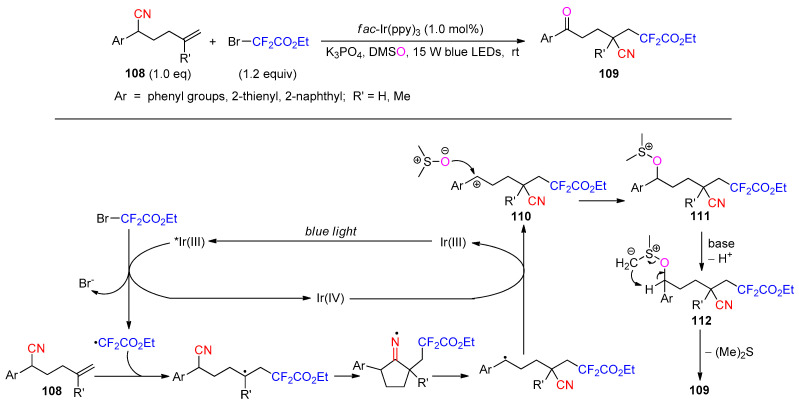
Photoredox functionalization of hexenenitriles with BrCF_2_CO_2_Et in MeCN (Guo, 2023) [93].

Guo described the photoredox conversion of 5-hexenenitriles **108** to difluoroalkylated ketonitriles **109** through the reaction with BrCF_2_CO_2_Et in DMSO using *fac-*Ir(ppy)_3_ as a catalyst and K_3_PO_4_ as a base (Scheme 31) [93], in which DMSO acts as both a solvent and a nucleophile, as in the formation of **106**. Once the carbocation intermediate **110** is formed via the oxidation of the benzylic radical, it will be captured via DMSO to give an alkoxysulfonium ion, **111**. Further deprotonation affords an alkoxysulfonium ylide, **112**, which then converts to the product through a [2,3]-sigmatropic shift.

**Scheme 32:**
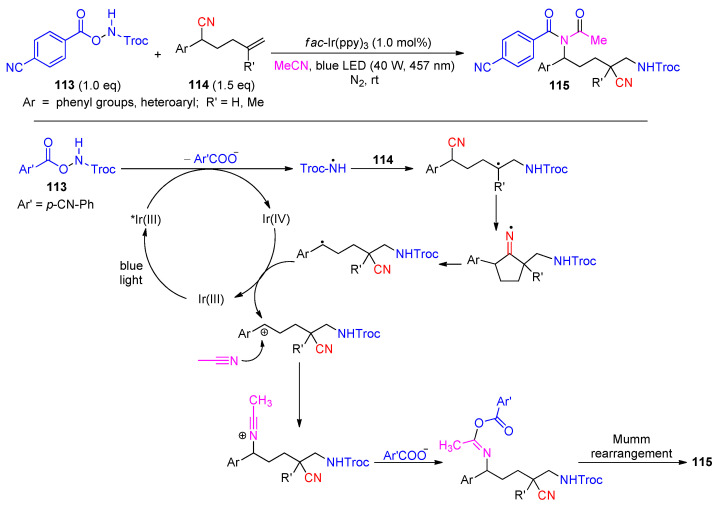
Photoredox diamidation of hexenenitriles using *O*-acyl hydroxylamine in MeCN (Akondi, 2025) [94].

More recently, Akondi et al. reported the employment of *O*-acyl hydroxylamines, such as **113**, as the bifunctional reagents to achieve the diamidation of 5-hexenenitriles **114** under photoredox catalysis. The reaction was conducted with 1 mol % of *fac*-Ir(ppy)_3_ in acetonitrile (as in the reaction of producing **105**) to afford the distal-imido β-amino nitriles **115** in varying yields (22 example, 21–81%) (Scheme 32) [94]. Mechanistically, the *O*-acyl hydroxylamine undergoes cleavage upon the single-electron reduction via the excited Ir(III) catalyst, affording a *N*-centered radical and a carboxylate anion. The 1,4-CN migration induced via the radical addition provides a benzylic radical that can be further oxidized into a carbocation by Ir(IV). The carbocation is then intercepted via acetonitrile to give a nitrilium ion. The attack of the nitrilium ion via the carboxylate anion combined with Mumm rearrangement furnish the desired product.

##### Reduction to Carbanions, Followed by Protonation or Nucleophilic Substitution

**Scheme 33:**
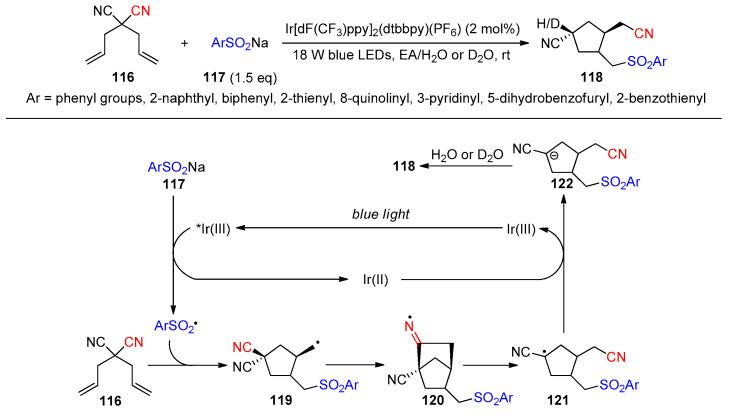
Construction of substituted cyclopentanes via radical-triggered transannular cyano-migration (Zhu, 2025) [95].

It has also been demonstrated that the carbon radicals resulting from cyano-migration can be in situ reduced to carbanions that are capable of undergoing protonation or nucleophilic substitution. Zhu and co-workers have revealed the construction of trisubstituted cyclopentanes **118** via the photoredox catalytic reaction of 4,4-dicyano-1,6-diene **116** with sodium (hetero)aryl sulfinates **117** in the presence of H_2_O or D_2_O (Scheme 33) [95]. Mechanistically, the sulfonyl radical generated from **117** under photoredox conditions could add to **116** to trigger a cyclization and produce cyclopentane intermediate **119**, bearing a primary alkyl radical. Intramolecular addition of the alkyl radical to the suprafacial cyano group affords the bicyclic iminyl radical **120**, which subsequently undergoes β-scission, yielding intermediate **121**. The β-scission is considered to be driven by the relief of ring strain, in addition to the radical stabilizing nitrile group [96]. The reduction in the alkyl radical in **121** via Ir(II) gives carbanion **122** with the regeneration of the Ir(III) catalyst. Finally, protonation or deuteration of **122** furnishes **118**. An array of sodium sulfinates containing various aryl and heteroaryl groups was employed for the reaction, and the desired products were all obtained as diastereomeric mixtures in about a 1:1 ratio (25 examples, 51–96%).

**Scheme 34:**
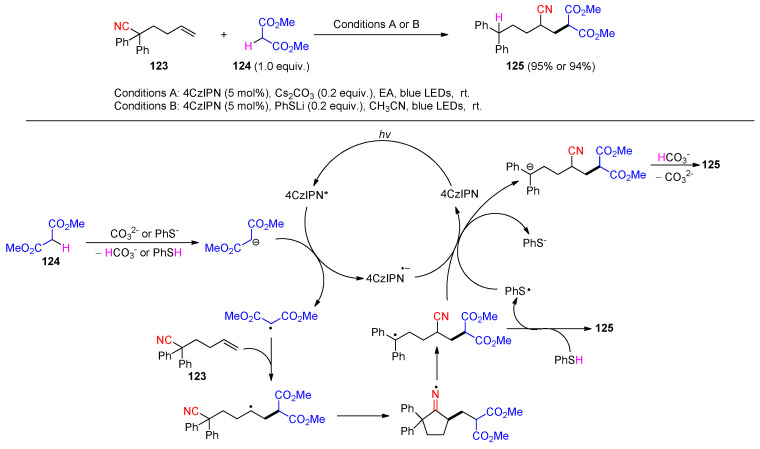
Alkylcyanation of unactivated Alkenes with protic C(sp^3^)-H feedstocks via cyano-group migration (Deng, 2025) [97].

Using photoredox/Brønsted–Lowry base dual catalysis, Deng et al. have developed a protocol for the alkylcyanation of unactivated alkenes with protic C(*sp*^3^)-H feedstocks via 1,4-cyano-group migration [97]. As described in the report, several mono- or diaryl-substituted 5-enenitriles were allowed to react with dimethyl malonate, triethyl methanetricarboxylate, methyl-Meldrum’s acid, methyl acetoacetate, or 1,3-cycloheptanedione in the presence of 2 mol% of 4CzIPN as the photocatalyst and 20 mol% of Cs_2_CO_3_ (Conditions A) or PhSLi (Conditions B) as base, leading to the generation of the alkylated nitrile products in varying yields (13 examples, 24–95%). The reaction of 2,2-diphenylhex-5-enenitrile **123**, with dimethyl malonate **124** yielding product **125**, can serve as an example to illustrate the mechanism (Scheme 34). The carbanion generated via the deprotonation of **124** is oxidized via the photoexited 4CzIPN to give a malonate radical. The addition of the radical to **123** triggers the cyano migration, leading to the benzylic radical intermediate that could be converted into **125** in two different ways. Under the conditions A, the benzylic radical may react with the reduced photocatalyst to deliver the benzylic anion. Further proton exchange with the conjugate acid furnishes **125** along with the base. Under the conditions B, the product is achieved directly from the benzylic radical through the hydrogen-atom transfer with PhSH. The reduction in the resulting phenyl sulfide radical by the 4CzIPN^•−^ species completes the photocatalytic cycle with the regeneration of phenyl sulfide anion. Ye et al. have recently demonstrated that the electrophilic alkyl radical shown in Scheme 34 could also be generated through the hydrogen-atom abstraction (HAA) of dimethyl malonate with an in situ-formed boryl radical under photocatalytic conditions, which then triggered the 1,4-CN migration of hexenenitrile derivatives and allowed the formation of alkene difunctionalization products after a further reduction, followed by protonation [98].

**Scheme 35:**
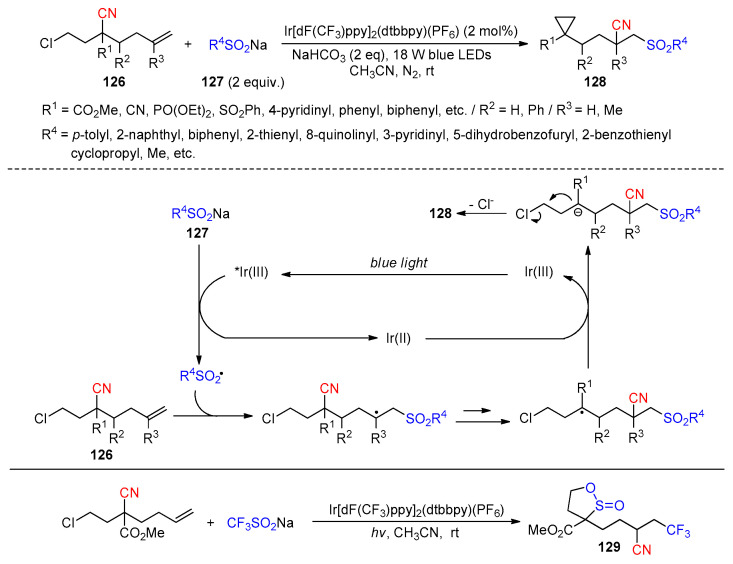
Cyano-migration-mediated radical–polar crossover cyclopropanation and formation of sultine product (Zhu, 2024) [99].

Zhu’s group disclosed a radical–polar crossover (RPC) reaction of 2-(2-chloroethyl)hex-5-enenitriles **126** with sodium sulfonates **127** under photocatalytic conditions, in which the carboanions generated through the reduction in the radical intermediates were shown to displace the terminal chloride to form multisubstituted cyclopropanes **128** (40 examples, 22–98%) (Scheme 35) [99]. Both aryl and aliphatic sodium sulfonates could be used for the reaction, while the compatibility with aliphatic sulfinates is particularly noteworthy, as they are susceptible to free radical desulfonylation under harsh conditions. The protocol also exhibited good tolerance to a wide range of functional groups present in **126** (R^1^), thus further enriching the diversity of the cyclopropane structures. Interestingly, when the reaction was conducted with Langlois’s reagent, the formation of a distinct sultine product **129** was observed through the insertion of SO_2_ during the cyclopropanation process.

##### Formation of CC Double Bonds via Oxidation/Deprotonation, Elimination, or HAT

**Scheme 36:**
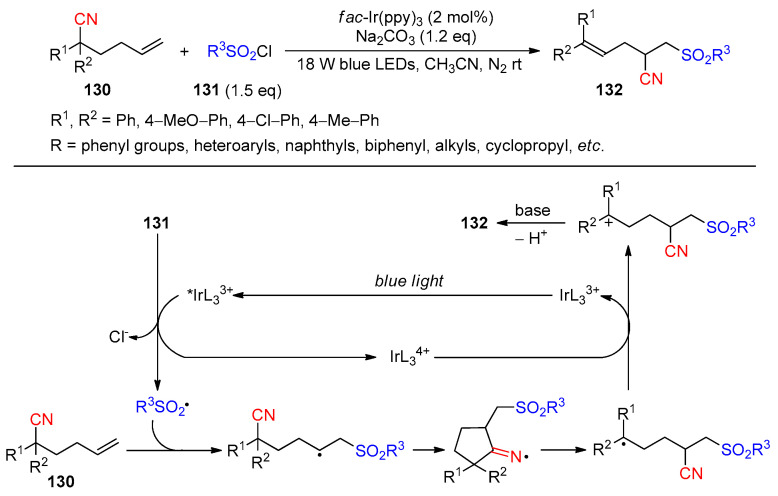
Formation of olefinic β-cyanosulfones via radical-mediated functionalization of diphenyl hexenenitriles (Zhu, 2023) [100].

After the cyano-group migration, the employed reaction conditions may enable the creation a carbon–carbon double bond through carbocation or radical or carbanion intermediates. Zhu and co-workers have developed a method allowing for the cyanosulfonylation of terminal alkenes with concurrent incorporation of a new C=C bond into the structure [100]. By using *fac*-Ir(ppy)_3_ as catalyst and Na_2_CO_3_ as a base, the photo-reaction of diphenyl hexenenitriles **130** with sulfonyl chlorides **131** afforded olefinic β-cyanosulfones **132** via consecutive sulfonylation, cyano-migration, single-electron oxidation of the benzyl radical, followed by deprotonation (26 examples, 35–88%) (Scheme 36). The reaction accommodates both aryl and aliphatic sulfonyl chlorides containing a broad range of substituents, but is not compatible to the alkyl-phenyl- or dialkyl-substituted hexenenitriles due to the weakened stability of radical or cation intermediates. Moreover, the products were obtained as the mixtures of *Z/E* isomers from unsymmetrically substituted substrates (R^1^ ≠ R^2^).

**Scheme 37:**
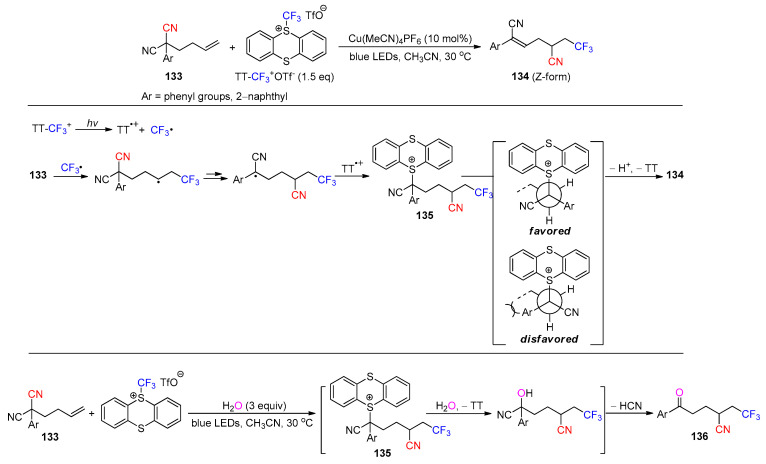
Photolytic reactions of alkenyl malononitriles with trifluoromethyl thianthrenium salt (Huang, 2023) [101].

Huang et al., in 2023, demonstrated the conversion of 2-aryl-2-(but-3-en-1-yl)malononitriles **133** into trifluoromethylated (*Z*)-alkenyl products **134** using trifluoromethyl thianthrenium triflate (TT-CF_3_^+^ OTf^−^) as a reagent and Cu(MeCN)_4_PF_6_ as a catalyst under the illumination of blue LED (19 examples, 49–82%) (Scheme 37) [101]. The reaction was carried out with phenyl- or 2-naphthyl-substituted substrates, which worked particularly well for those possessing *para*-electron-donating groups at the phenyl ring. It is postulated that TT-CF_3_^+^ can fragment into TT^·+^ and trifluoromethyl radical upon the absorption of blue light. The addition of the CF_3_ radical to the terminal olefin, followed by 1,4-cyano-migration, leads to a tertiary radical intermediate, which is captured via TT^·+^ to afford an α-thianthrenium cyano species **135**, as proven by high-resolution mass spectrometry. Intermediate **135** then undergoes a *trans*-elimination, possibly with the assistance of the copper catalyst, to provide the product and release TT. The remarkable *Z*-selectivity observed in this reaction is rationalized through the conformation adopted for the *trans*-elimination, in which the steric interaction between the bulky aryl group and alkyl chain can be significantly minimized. Furthermore, the addition of 1 mol % of Ir[dF(CF_3_)ppy]_2_(dtbbpy)PF_6_ to the reaction mixture was shown to improve the yield of the desired product (Ar = Ph, 93% vs. 70%), but it resulted in poorer diastereoselectivity (*Z/E* ratio = 3:1) due to an alkene isomerization enabled via the energy-transfer photocatalyst. The report also revealed that the reaction pathway of intermediate **135** was altered in the presence of water, yielding the ketone products **136** via a nucleophilic substitution process (12 examples, 48–77%).

**Scheme 38:**
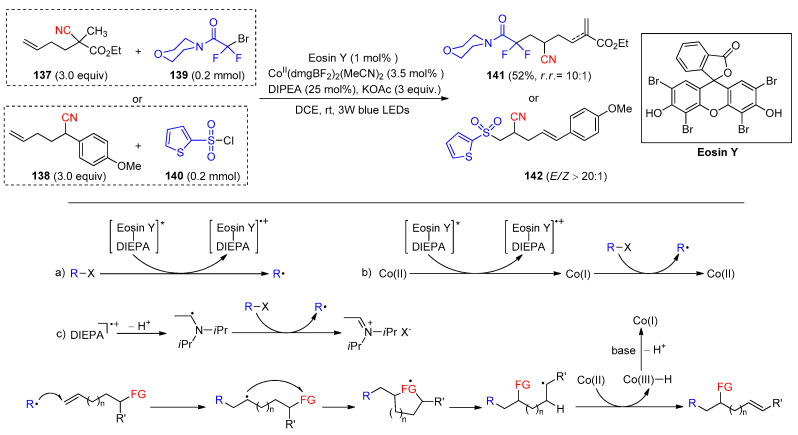
Photo/cobalt-catalyzed functionalization of alkenes via FGM and co-promoted HAT (Wang, 2024) [102].

Wang and co-workers have developed an alternative method for achieving alkene difunctionalization with the simultaneous creation of a C-C double bond via photo-induced functional group migration (FGM) and Co-promoted C(*sp*^3^)-H desaturation [102]. It shows good compatibility with diverse fluoroalkyl and sulfonyl radical precursors, as well as alkenes containing the benzoyloxy, acetoxy, formyl, heteroaryl, or cyano-migration group. In the study, ethyl 2-cyano-2-methylhex-5-enoate **137** and several phenyl-substituted 5-enenitriles, e.g., **138**, were investigated for the 1,4-cyano-migration using 2-bromo-2,2-difluoroacetamides and sulfonyl chlorides such as **139** and **140** as radical precursors. The reaction was performed with 1 mol% Eosin Y as a photocatalyst, 3.5 mol% Co^II^(dmgBF_2_)_2_(MeCN)_2_ as an HAT catalyst (dmg = dimethylglyoxime), 25 mol% DIPEA as a ligand, and 3 equiv. of KOAc as a base in a DCE solution under blue LED illumination, which provided a total of 20 products with a remarkable site- and stereo-selectivity (*E*:*Z* > 20:1), as exemplified by **141** and **142** (Scheme 38).

A general pathway has been proposed based on mechanistic studies and DFT calculations. There may be several ways to generate radicals from the precursors R-X to trigger the reactions. Ultraviolet-visible and other experiments have suggested the formation of an electron donor–acceptor complex between Eosin Y and DIPEA, which could react with R-X on its excited state to form the radicals (a) [103]. Another is the reduction in cobaloxime(II) via the complex to Co(I), which then leads to radical generation through a halogen-atom transfer (XAT) (b). Moreover, DIPEA may act as a reducing agent to yield an aminoalkyl radical after an oxidative deprotonation. Thus, the pathway of XAT activation of R-X by an aminoalkyl radical is also possible (c) [104]. The resulting radicals then trigger the FGM through the addition to olefins, yielding thermodynamically more stable radicals. With the formation of these carbon-centered radicals, the adjacent C(*sp*^3^)-H bonds can be effectively weakened, allowing for a cobaloxime(II)-assisted HAT process to give the products.

**Scheme 39:**
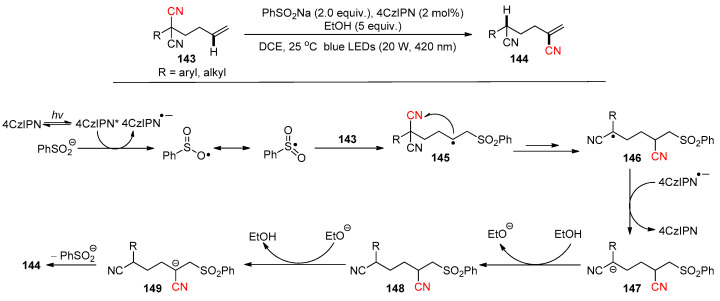
Branch-selective cyanation of alkenes through photo-induced traceless functional group translocation (Shu, 2025) [105].

In the aforementioned cases, the CC double bonds were created at the sites where the CN groups were originally attached. Very recently, Shu et al. reported the first branch-selective cyanation of alkenes via visible-light-mediated traceless functional group translocation, allowing the conversion of a range of malononitrile-substituted terminal butenes **143** into the corresponding α,β-unsaturated nitrile derivatives **144** under the conditions shown in Scheme 39 (32 examples, 40–95%) [105]. In addition to sodium benzenesulfinate as radical source and 4CzIPN as photocatalyst, ethanol was employed as an important additive to improve reaction efficiency. Based on DFT calculations and the results from control experiments, a plausible mechanism has been proposed for the transformation. First, photoexcitation of 4CzIPN induces a single-electron transfer (SET) with the sulfite anion, generating a sulfite radical and its sulfur-centered resonance structure. This reactive species then adds to **143**, leading to intermediate **145** and further **146** via the CN migration. The SET between **146** and 4CzIPN^•−^ generates the carbon anion **147** that can be protonated under ethanol-rich conditions to provide the β-cyanosulfone **148** and EtONa in situ. Deprotonation of **148** produces carbon anion **149**, which subsequently undergoes elimination to yield the target product.

#### 2.3.5. Recently Reported Cyano Migrations Involving Diradical or Radical Cation Intermediates

**Scheme 40:**
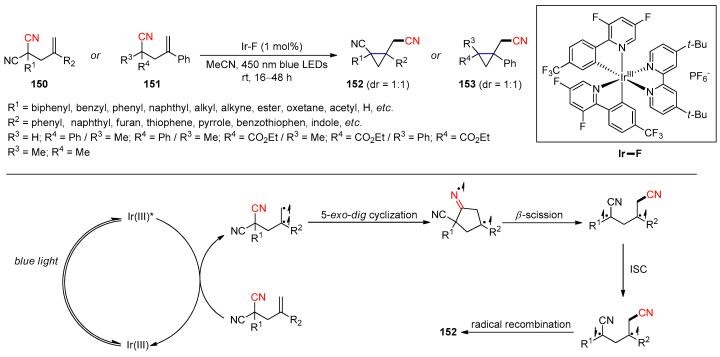
Di-π-ethane rearrangement of cyano groups via energy-transfer catalysis (Huang, 2024) [106].

Recent advancements in photochemistry have led to the development of several strategies to achieve cyano-migration without altering the overall composition of alkenyl nitrile substrates (100% atom economy). Huang and co-workers, in 2024, reported the rearrangement of 2-allylmalononitriles **150** or 4-pentenenitriles **151** into the cyclopropane products **152** or **153** by using 1 mol% Ir-F as a photocatalyst in CH_3_CN under blue-light irradiation (Scheme 40) [106]. They proposed that a diradical intermediate could be generated from the C-C double bond through an energy-transfer mechanism facilitated by photoexcited iridium-based photocatalysis. Subsequent 5-exo-dig cyclization, followed by the β-scission of the resulting imido radical, provides another diradical intermediate. After intersystem crossing (ISC), the newly formed intermediate may undergo a radical-radical recombination to afford the three-membered-ring products. This unprecedented approach was defined as a di-π-ethane rearrangement in order to differentiate from the known di-π-methane rearrangement (Zimmerman rearrangement), wherein two π systems engaging in photochemical rearrangement are separated by one *sp*^3^ carbon atom (methane-like). Over fifty substrates **150** containing various R^1^ and R^2^ substituents, as well as the indicated **151**, could undergo the reaction smoothly to deliver the products in good to excellent yields (48–98%, mostly >90%). In a few exceptional cases, the desired products were not obtained from malononitriles bearing tri- or tetrasubstituted alkenyl groups and 4-pentenenitriles containing hydrogen-atom(s) or a piperidinyl ring [R^3^ = R^4^ = H; R^3^ =Me/R^4^ = H; R^3^ = CO_2_Et/R^4^ = H or R^3^ = R^4^ = -CH_2_CH_2_N(Boc)CH_2_CH_2_-], probably due to the altered reactivity of in situ-formed radical species or steric hindrance. The author also demonstrated that this protocol was not applicable to the translocation of cyano group through a three- or six-membered-ring transition state. Shortly after this report, Zhong et al. described a similar method for the cyclopropanation via the migration of the cyano and aryl functional groups using 0.01 to 1 mol% 2-chlorothioxanthone as a photosensitizer [107].

**Scheme 41:**
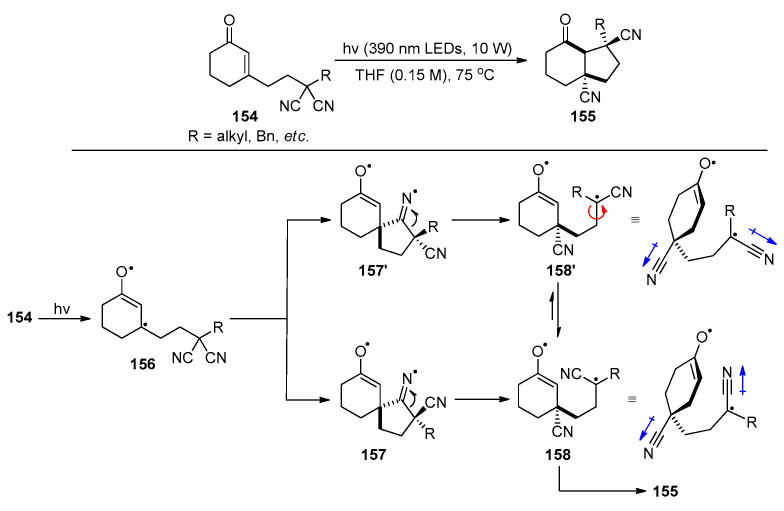
Diastereoselective synthesis of 5/6fFused bicyclic ring systems via radical cyano-group migration (Wang, 2025) [108].

The diradical intermediate can also be formed with a cyclic enone chromophore, as suggested by Wang’s group, who recently reported the radical cyclization of 3-substituted cyclohexenone derivatives **154** into 5/6-fused bicyclic compounds **155** via cyano-group transfer under catalyst-free and near-ultraviolet light irradiation conditions (22 examples, 67–99%, up to > 20:1 d.r.) (Scheme 41) [108]. It has been suggested that the direct exposure of **154** to near-UV light could trigger the nπ* excitation to provide a triplet diradical intermediate **156** through intersystem crossing (ISC). The intramolecular radical addition of the tertiary alkyl radical to either cyano group results in the formation of imine radical **157** and its diastereomer **157′**. Subsequent β-scission facilitates cyano-group transfer to yield diradical intermediate **158** and its conformational isomer **158′**, which could interconvert readily through a facile σ-bond rotation. The diastereoselectivity observed in the reaction should be governed by the conformational distribution of **158** and **158′**, while **158** is considered to be more favorable than **158′** due to the opposite orientation of the cyano groups to mitigate dipole–dipole repulsion as well as the π-stacking interaction between the cyano group and the α-carbonyl radical to render it more stable. Intermediate **158**, upon reverse intersystem crossing (RISC) between the triplet and singlet states, rapidly undergoes a cyclization on its singlet state to afford the products. This reaction is also applicable to ester/nitrile-substituted cyclohexenones or dinitrile-substituted cyclopentenones but giving the desired products in lower yields and/or diastereoselectivity.

**Scheme 42:**
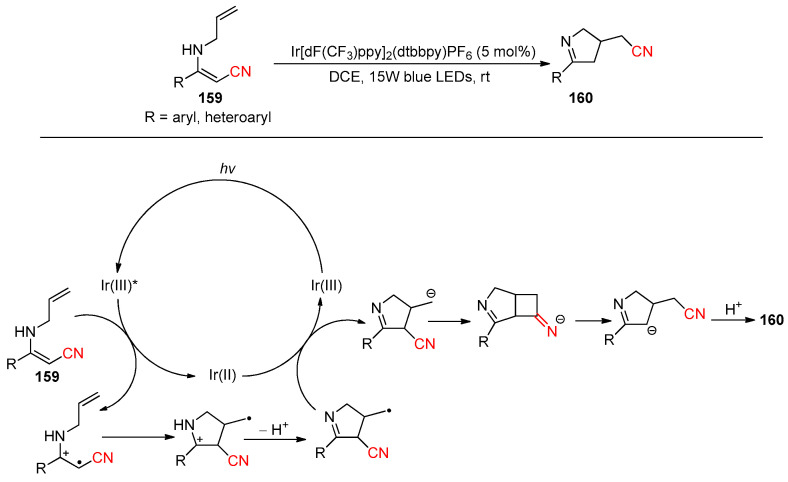
Synthesis of cyclic imines via radical-triggered 1,3-cyano migration of *N*-allyl enamines (Pan, 2024) [109].

The earlier reported 1,3-cyano-migration reactions generally required elevated temperatures to overcome the energy barrier associated with highly strained four-membered ring intermediates (Scheme 6, Scheme 7, Scheme 8 and Scheme 12). Pan et al. recently reported a visible light-induced tandem cyclization/1,3-cyano-migration process, enabling the conversion of nitrile-substituted *N*-allyl enamines **159** into the corresponding cyclic imines **160** under relatively mild conditions (26 example, 44–93%) (Scheme 42) [109]. Mechanistically, the reaction may proceed through a SET oxidation of **159** via a photoexcited Ir(III) catalyst to generate a radical cation intermediate. Subsequent 5-exo-trig cyclization, followed by deprotonation, affords cyclic imine bearing a methylene radical. This intermediate could be further reduced into a carbanion by Ir(II) species. From this, the products can be formed via sequential nucleophilic addition, ring opening, and protonation. Moreover, a 1,3-acyl migration can be realized when the cyano moiety in **159** is replaced with an ester group.

#### 2.3.6. Application of CN Migration in Polymerization

**Scheme 43:**
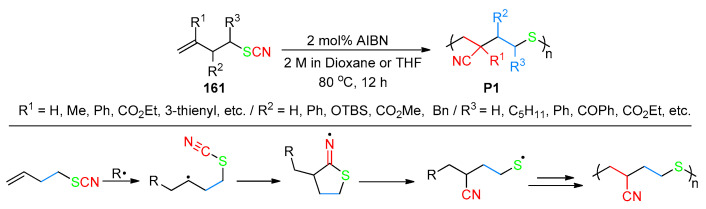
Homopolymerization of linear α-olefins enabled by radical-mediated 1,4-cyano-group migration (2024, Li) [112].

The radical addition-triggered cyano-group migration has also been applied to polymer synthesis [110,111,112,113]. It is well accepted that α-olefins and their functionalized derivatives are nearly impossible to undergo homopolymerization under radical conditions due to the interference from chain-transfer side reactions. For instance, the highly reactive propagating secondary carbon-centered radicals, instead of adding to the double bond, tend to abstract an allylic hydrogen atom from another monomer to generate more stable allylic radicals. To cope with this problem, Li et al. have recently reported a study on the homopolymerization of thiocyanate functionalized linear α-olefins enabled via radical-mediated 1,4-cyano transfer [112]. A vast array of monomers **161** with different functionalities could undergo polymerization, allowing access to a library of ABC sequence-regulated polymers **P1** with high molecular weights and unprecedented structures (Scheme 43). For instance, the secondary carbon radical generated from 4-thiocyanato-1-butene after the initiation step undergoes a 5-exo-dig onto the cyano group. Subsequent β-fragmentation of the iminyl radical provides a relatively stable thiyl radical, which then adds to another monomer and so on to eventually lead to the polymerized product [Conv. > 95%; M_n_ = 28.7 k (Dioxane), 20.3 k (THF)]. In another noteworthy report, Sun et al. revealed the preparation of ABC sequence-defined polymers from the corresponding hept-6-enenitriles via radical-mediated 1,5-cyano-migration [113].

### 2.4. Cyano-Group Migrations via Halogen-Atom Transfer

**Scheme 44:**
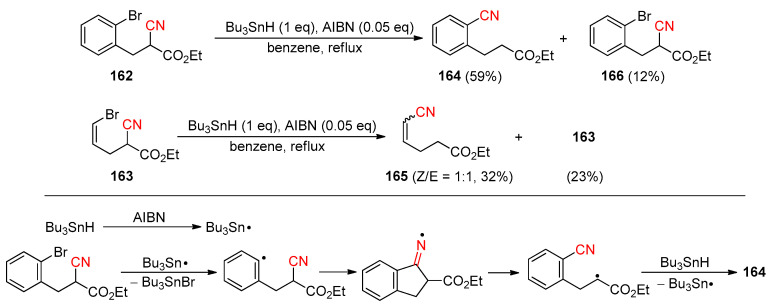
XAT-mediated CN migration of *o*-bromophenyl and bromovinyl cyanoacetates (Beckwith, 1987, 1988) [114,115].

Halogen-atom transfer (XAT) between carbon-halogen bonds and tributyltin radical has long been known as an approach to generate carbon-centered radical to promote cyano-migration. In the earlier reports (1987 and 1988), Beckwith demonstrated that heating of cyanoacetate **162** or **163** with *n*-Bu_3_SnH and AIBN initiator in refluxing benzene could produce the l,4-transfer products **164** and **165**, along with the simple dehalogenation product **166** or recovered starting material (Scheme 44) [114,115]. The probable route for the translocation involves the cyclisation of the initially formed aryl (or vinyl) radical onto the nitrile to give a cyclic iminyl radical. This intermediate undergoes rapid β-scission to yield **164** after hydrogen-atom abstraction. In these cases, the β-scission is aided by the presence of a radical-stabilizing ester substituent.

**Scheme 45:**
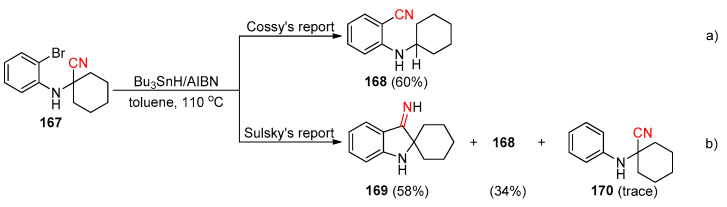
Reactions reported for α-(bromophenylamino)nitrile **146** under the same conditions (**a**) Cossy’s report. (**b**) Sulsky’s report (Cossy, 1995; Sulsky, 1999) [116,117].

**Figure 3:**
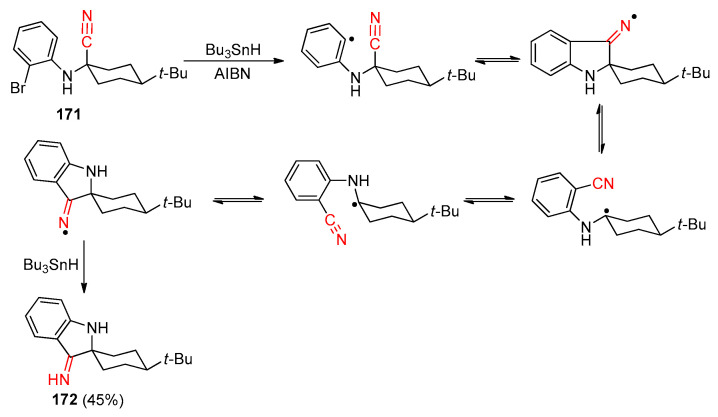
Configuration inversion during the cyclization of **150** into **151** (Sulsky, 1999) [117].

About a decade later, Cossy et al. showed that the similar nitrile transfer reaction could proceed in moderate yields with α-(bromophenylamino)nitriles such as **167** upon the treatment with Bu_3_SnH/AIBN in refluxing toluene, affording 2-(alkylamino)benzonitriles such as **168** as the only isolated products (Scheme 45a) [116]. However, a report by Sulsky four years later described a very different outcome from those reported by Cossy [117]. For example, the same treatment on **167** led to the spiroindoxylimine **169** as the major product (58%), together with a 24:1 mixture of **168** and the reduction product **170** in 35% yield (Scheme 45b). They speculated that the highly polar **169** could not have been isolated under the chromatography conditions described by Cossy. Through single-crystal X-ray analyses, Sulsky also discovered that the relative configuration of the anilino nitrogen in precursor **171** (equatorial/trans to the *tert*-butyl group) had been inverted during the cyclization to spiroimine derivative **172** (axial/cis to the *tert*-butyl group). This suggests that the ring opening and configuration inversion, followed by ring closure, must occur rapidly in a reversible fashion and that the cis isomer is more stable than the trans isomer (Figure 3).

**Scheme 46:**
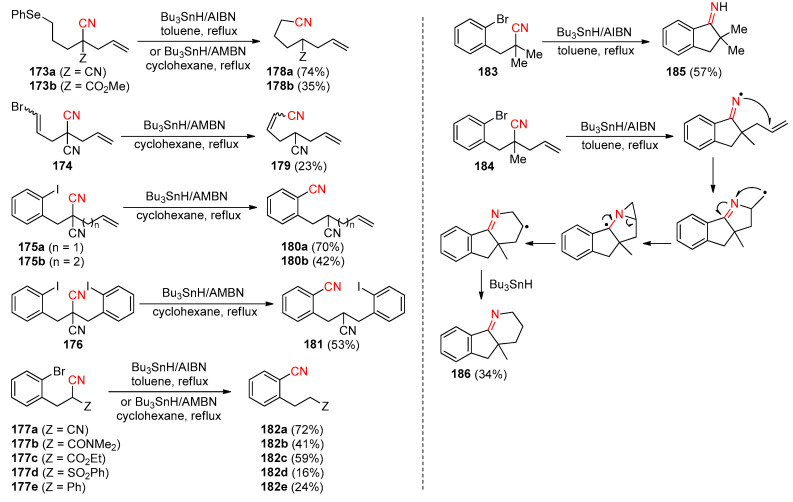
Nitrile transfer or cyclization reactions influenced by α-substituents (Bowman, 2000) [118].

The aforementioned results suggest that the reactivity of intermediate iminyl radicals, generated via the radical cyclisation onto nitrile groups, could be influenced by α-substituent, i.e., ester in Scheme 44 and amino in Scheme 45 and Figure 3. A study carried out by Bowman et al. has further validated this concept by using a set of phenylselenyl, bromo, and iodo radical precursors [118]. With the presence of α-CN, CO_2_R, CONMe_2_, SO2Ph, or Ph substituent, the iminyl radicals derived from **173**–**177** tended to undergo β-scission to yield stable/irreversible ring-opened radicals and, thus, the translocation products **178**–**182** (Scheme 46). Note that unusual C-Se bond cleavage was utilized to generate the alkyl carbon radicals to perform the addition with the nitriles in the production of **178**. When the α-substituent was an alkyl group, the formation of nitrile transfer products was not observed but, rather, cyclised imines or a triheterocycle derivative, as is shown with the conversion of **183** and **184** into **185** and **186**.

**Scheme 47:**
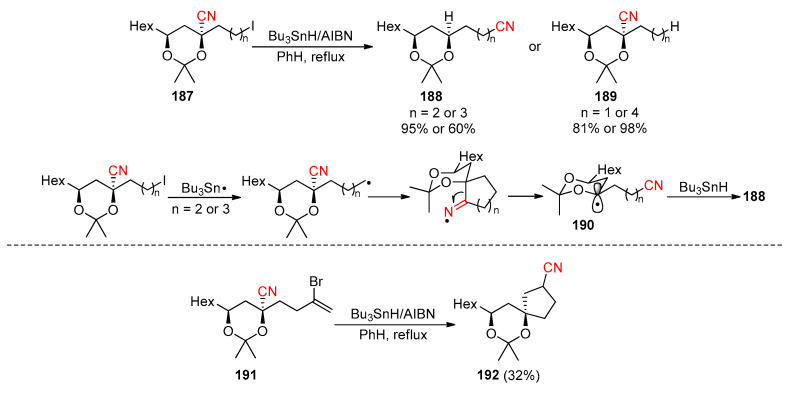
XAT-mediated cyano-migration of 1,3-dioxane-4-nitriles (Ryclmovsky, 1997) [119].

For 1,3-dioxane-4-nitriles **187**, Ryclmovsky demonstrated that the 1,4- and 1,5-nitrile transfer reactions (*n* = 2, 3) could take place efficiently to give the products **188**, whereas the corresponding 1,3- and 1,6-nitrile transfer reactions (*n* = 1, 4) were not observed and instead led to the dehalogenation products **189** (Scheme 47) [119]. During the nitrile transfer, the ring opening of the iminyl radical should be encouraged through the formation of the stabilized α-alkoxy radical. Moreover, products **188** were exclusively obtained as the *syn*-l,3-diol isomers, which is presumably a result of the stereoselective reduction in intermediate **190** with Bu_3_SnH. The authors also revealed that the α-alkoxy radicals produced in nitrile transfer could participate in a cyclization with appropriate unsaturated substituents. For instance, the treatment of vinyl bromide **191** with Bu_3_SnH and catalytic AIBN in refluxing benzene provided the spirocyclic compound **192** via a tandem transfer-cyclization process. Although a 5-endo-trig cyclization like this one is generally disfavored according to Baldwin’s rules, the complementary electronic distributions of the α-alkoxy radical and β-cyano alkene are supposed to favor cyclization.

**Scheme 48:**
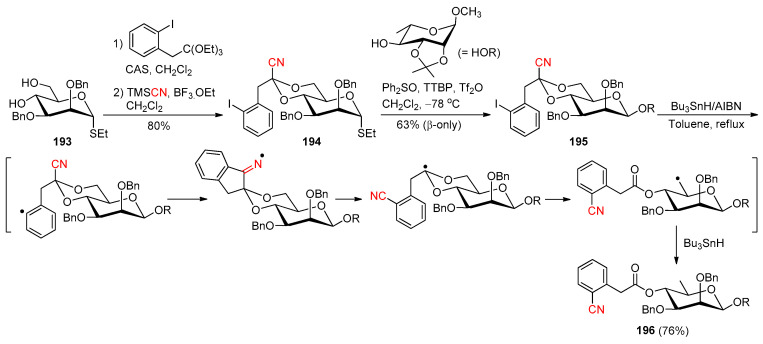
Preparation of β-rhamnopyranoside from thioglycoside via nitrile transfer/fragmentation (Crich, 2006) [120].

**Scheme 49:**
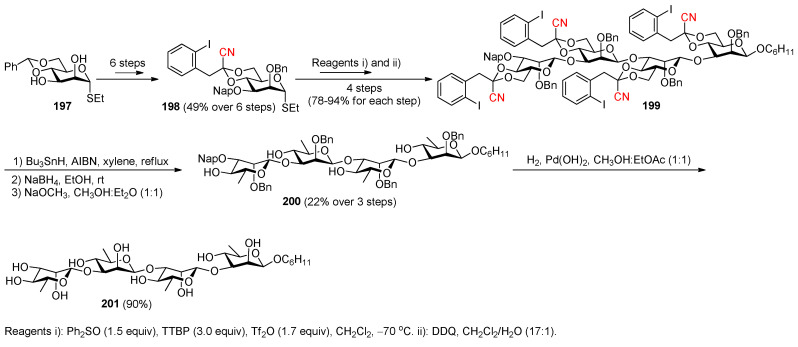
Total synthesis of a natural tetrasaccharide via one-pot quadruple radical fragmentation (Crich, 2006) [121].

The strategy described in this section has been utilized in the syntheses of complex and biologically important natural molecules. Crich and Bowers reported a method for the preparation of β-rhamnopyranosides from thioglycosides, as exemplified by the conversion of **193** into **196** (Scheme 48) [120]. In the sequence, the 4,6-*O*-[1-cyano-2-(2-iodophenyl)-ethylidene] group was first introduced to **193** as a single diastereomer via the acid-catalyzed reaction of the 4,6-diol with triethyl (2-iodophenyl)orthoacetate, followed by BF_3_·OEt_2_-promoted cyanation. The acetal-protecting group in the resulting product **194** conveys strong β-selectivity to allow the stereocontrolled formation of β-mannoside **195** through the coupling with methyl 2,3-O-isopropylidene-*R*-*L*-rhamnopyranoside. As a key operation, treatment of **195** with tributyltin hydride and AIBN in toluene at reflux triggered a cyano transfer/fragmentation process to produce the product **196**. The utility of this method was further demonstrated by the concise synthesis of a naturally occurring β-(1→3)-D-rhamnotetraose via a one-pot quadruple radical fragmentation [121]. To this end, a suitably protected monomer **198** was first synthesized from 4,6-*O*-benzylidene-protected thiomannoside **197** in six steps (Scheme 49). After this, the sequential coupling reactions of **198** were carried out, first with cyclohexanol and then with the 3-OH-deprotected oligomer formed in each step, to cause the formation of a β-mannotetraose **199**. The subjection of **199** to conditions of radical fragmentation in refluxing xylenes, followed by NaBH_4_ reduction to facilitate the removal of tin residues and then saponification, provided the precursor tetraol **200**. Finally, the deprotection of **200** via hydrogenation eventually furnished the target molecule **201** at a 90% yield.

**Scheme 50:**
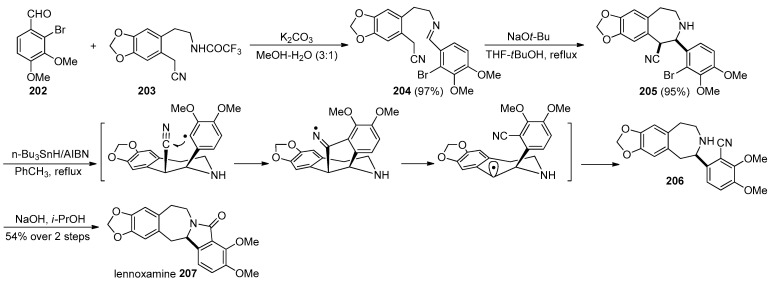
Synthesis of lennoxamine through cyano-migration (Orito, 2009) [122].

Orito and co-workers reported the total synthesis of a natural isoindolobenzazepine alkaloid, lennoxamine, as illustrated in Scheme 50 [122]. The condensation of bromobenzaldehyde **202** with trifluoroacetamide **203** produced imine **204**, which underwent cyclization under the basic conditions to yield benzazepine **205**. The further treatment of **205** with Bu_3_SnH/AIBN under thermal conditions yielded the 1,4-CN migration product **206**. The base-promoted cyclization of **206** then furnished lennoxamine **207**.

### 2.5. Cyano-Group Migrations via C-C Bond Fragmentation

Radical-mediated C-C bond fragmentation can be used as another approach for producing the carbon-centered radical to trigger cyano-migration, yet it has only been limited to a few reports.

**Scheme 51:**
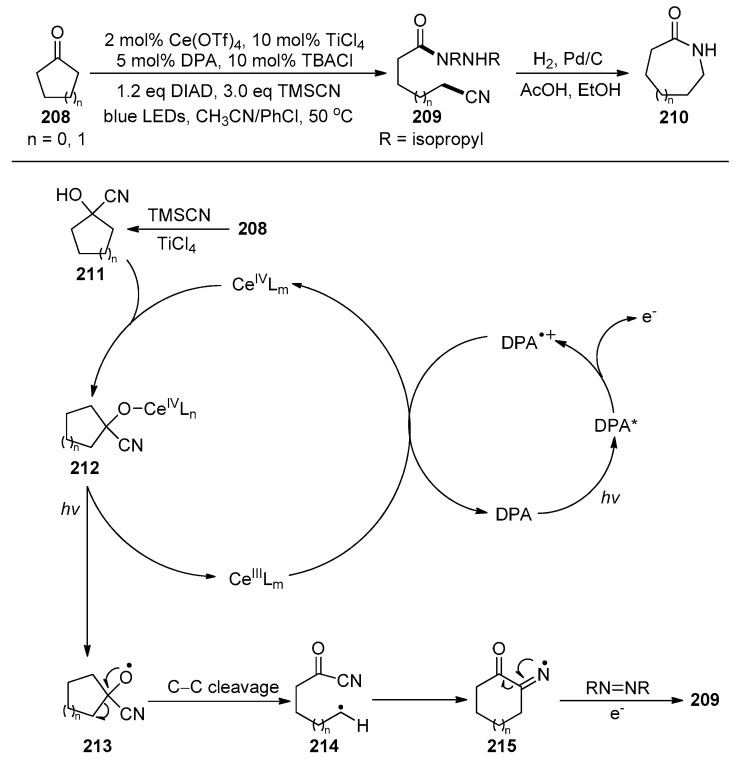
Functionalization of cyclic ketones via LMCT-mediated C-C bond cleavage and cyano-migration (Zou, 2020) [123].

Zuo and colleagues exploited the selective C-C bond cleavage of ketones through a cooperative utilization of Lewis acid catalysis and ligand-to-metal charge transfer (LMCT) excitation [123]. In their report, both cylobutanone and cyclopentanone derivatives **208** were converted to the corresponding cyanated hydrazides **209** under the conditions shown in Scheme 51 (18 examples, 51–97%), which could serve as precursors to the preparation of lactam **210** via a simple hydrogenation operation.

The proposed pathway involves the generation of cyanohydrin **211** via the nucleophilic addition with TMSCN. In addition to cerium triflate, the titanium tetrachloride catalyst was required in this step to convert inactive cyanohydrin silyl ether into cyanohydrin. Under blue-light irradiation, the cerium(IV)-cyanohydrin coordination complex **212** can be excited to promote the homolysis of the Ce-O bond, generating an alkoxy radical **213**. The subsequent cleavage of the α-C-C bond produces intermediate **214** bearing a carbon-centered radical, which then adds to the cyano group to form carbonyl imino radical **215**. The trapping of **215** with diisopropyl azodicarboxylate (DIAD), followed by a photo-induced single-electron reduction with 9,10-diphenylanthracene (DPA), provides the products **209**, along with a radical cation of DPA. The radical cation then engages in single-electron transfer with cerium(III) to complete the catalytic cycle. In the cases of α-substituted cyclic ketones, the C-C bond between the most substituted α-carbon and the carbonyl group was selectively cleaved to form a more stable carbon radical. Moreover, the radical intermediates derived from cyclohexanones (or ring systems larger than six-membered) and acyclic ketones did not undergo cyano-migration after the C-C bond cleavage but instead reacted with DIAD and introduced methanol to afford aminated carboxylic esters as the products.

**Scheme 52:**
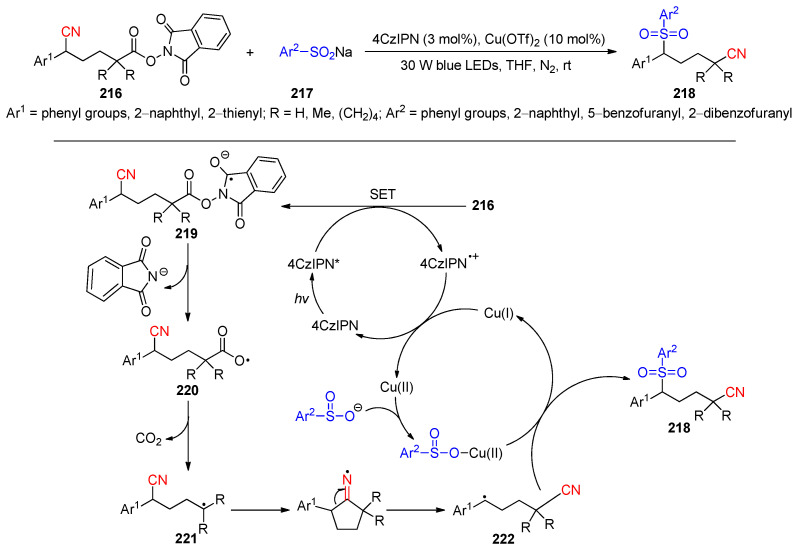
Preparation of δ-sulfonyl nitriles via decarboxylation-triggered 1,4-cyano-migration (Chen, 2024) [124].

A photoredox/Cu dual-catalyzed 1,4-cyanosulfonylation of *N*-hydroxyphthalimide esters **216** with sodium arylsulfinates **217** has been established by Chen et al., which provides a variety of δ-sulfonyl nitriles **218** via a decarboxylation-triggered remote cyano-migration under the conditions shown in Scheme 52 (30 examples, 30–72%) [124]. In the proposed mechanism, the SET reduction in **216** through the excited photocatalyst 4CzIPN* takes place to produce intermediate **219** and the 4CzIPN radical cation. The elimination of the imide anion from **219** via N-O bond cleavage gives the carboxyl radical **220**, which subsequently undergoes a decarboxylation yielding intermediate **221**, bearing an alkyl radical to trigger the 1,4-cyano-migration. Afterwards, the resulting benzylic radical **222** could be trapped by the sulfonate radical released from the in situ-formed Cu(II)-arylsulfinate complex to furnish the products **218**. The Cu(I) generated in this step can be re-oxidized through the 4CzIPN radical cation, leading to both Cu(II) and 4CzIPN catalysts.

**Scheme 53:**
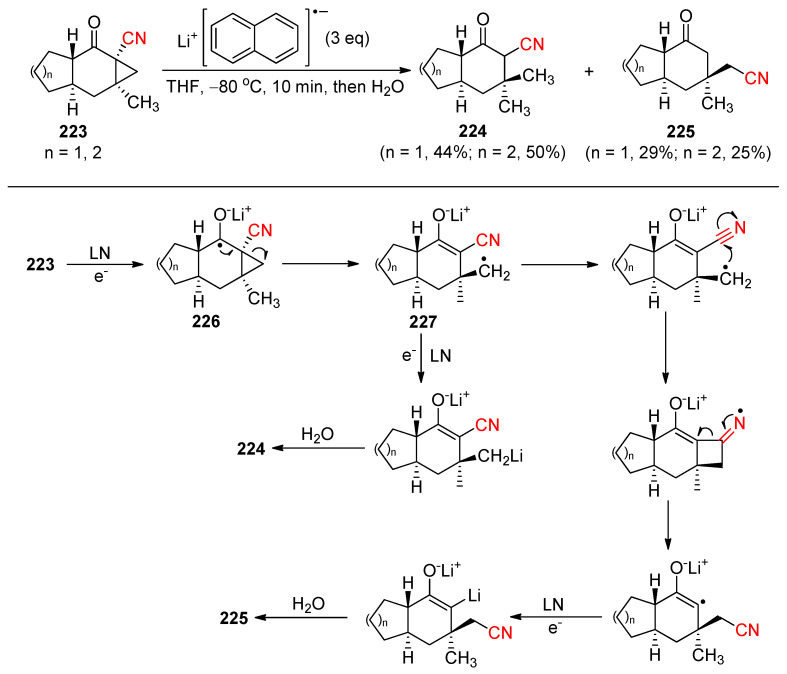
1,3-Cyano migration triggered by reductive ring opening (Zhu, 2017) [125].

We previously synthesized the fused tricyclic α-keto cyclopropyl nitriles **223** via the rhodium-catalyzed intramolecular cyclopropanation. The treatment of **223** with lithium naphthalenide (LN) as an electron-donating agent produced α-cyano ketones **224** as the major products, along with γ-cyano ketones **225** resulting from an additional 1,3-cyano-migration (Scheme 53) [125]. We proposed that **223** was first reduced to ketyl radical anions **226** via LN, which subsequently underwent the ring opening yielding radical anion intermediates **227**. The carbon radicals in **227** could cyclize onto the nitrile group to cause the formation of **225** after β-scission, reduction, and protonation. Moreover, the very carbon radicals could also be reduced into carbanions in the presence of excess of LN to afford **224**. From the yields of the products, it is reasonable to say that the reduction in the carbon radicals should occur faster or more easily than the addition to the nitrile group. Moreover, the exclusive formation of α-cyano ketones was observed when the angular methyl groups in **223** were replaced with a hydrogen atom, implying that the methyl groups might play a role in directing the reaction pathway, possibly through steric effects.

## 3. Conclusions

The radical-mediated cyano-group translocation is a crucial strategy in organic synthesis, enabling the precise customization of molecular structures and leading to the creation of a wide array of important molecules, as discussed throughout this paper. It has also found application in the total synthesis of natural products (Scheme 49 and Scheme 50), as well as in material science (Section 2.3.6). Mechanistically, the process requires the initial generation of a carbon-centered radical to trigger the CN migration and provide a more stable carbon radical for further elaboration. Decades of studies on this topic have led to the discovery of a plethora of procedures to achieve migration, which are systematically reviewed in four sections, according to the strategies used to generate the initial carbon radicals. As we have shown, the earlier reported experiments were mostly conducted under harsh thermal conditions to give the products in low yields. Moreover, a great number of mild and efficient methods have been reported over the past twenty years with advancements in radical chemistry and photocatalysis. Particularly remarkable is the diversity of photocatalytic techniques that have been used for the method development, such as photoredox catalysis, organo-photocatalysis, NHC-assisted photoredox catalytsis, dual metal/photoredox catalysis, and photo-induced ligand-to-metal charge transfer (LMCT), which allow the generation of reactive intermediates under mild reaction conditions, thus facilitating the CN migration and/or the incorporation of other functional group(s) into molecules. Predictably, the search for novel and efficient reagents, as well as the target-based design of nitrile substrates, will continue to be the focus of future investigation, together with mechanistic studies for some newly discovered processes. Overall, the article provides a comprehensive assessment of all cases reported for radical-mediated cyano-group migration, and it may serve as a guide for research in this particular field.

## Data Availability

No new data were created or analyzed in this study. Data sharing is not applicable to this article.
